# Dynamics of tryptophan metabolites and microbial adaptations during corn by-product fermentation in the pig gut microbiome

**DOI:** 10.1186/s40104-026-01364-4

**Published:** 2026-02-18

**Authors:** Eita Toyoshi, Masahiro Watanabe, Fu Namai, Kenji Yamane, Toma Kashima, Wakako Ikeda-Ohtsubo, Afifah Zahra Agista, Ayu Yoshida, Taiga Sakuma, Itsuko Fukuda, Kasumi Suzuki, Gou Yoshioka, Yuji Imai, Sae Tsuchida, Eri Nishiyama, Hiroki Shinkai, Yoshihiro Muneta, Hirohide Uenishi, Ryuta Tobe, Hitoshi Shirakawa, Masamitsu Maekawa, Nariyasu Mano, Haruki Kitazawa, Keita Nishiyama

**Affiliations:** 1 Food and Feed Immunology Group, Laboratory of Animal Food Function, Graduate School of Agricultural Science, Tohoku University, Sendai, 980-8572 Japan; 2 Graduate School of Pharmaceutical Sciences, Tohoku University, Sendai, 980-8574 Japan; 3 Livestock Immunology Unit, International Education and Research Centre for Food and Agricultural Immunology (CFAI), Graduate School of Agricultural Science, Tohoku University, Sendai, 980-8572 Japan; 4 Nihon Shokuhin Kako Co., Ltd., 30 Tajima, Fuji, Shizuoka, 417-8530 Japan; 5 Department of Biotechnology, The University of Tokyo, Bunkyo-Ku, Tokyo, 113-8657 Japan; 6 Laboratory of Nutrition, Graduate School of Agricultural Science, Tohoku University, Sendai, 980-8572 Japan; 7 Department of Agrobioscience, Graduate School of Agricultural Science, Kobe University, Nada-Ku, Kobe, Hyogo 657-8501 Japan; 8 Swine and Poultry Research Department, Gifu Prefectural Livestock Research Institute, Seki, 501-3924 Japan; 9 Miyagi Prefecture Animal Industry Experiment Station, Osaki, Miyagi 989-6445 Japan; 10 Biotechnological Research Support Division, FASMAC Co., Ltd., Atsugi, 243-0041 Japan; 11 National Institute of Animal Health, National Agriculture and Food Research Organization, Tsukuba, 305-0856 Japan; 12 Institute of Agrobiological Sciences, National Agriculture and Food Research Organization, Tsukuba, 305-8634 Japan; 13 Laboratory of Animal Microbiology, Graduate School of Agricultural Science, Tohoku University, Sendai, 980-8572 Japan; 14 Department of Pharmaceutical Sciences, Tohoku University Hospital, Sendai, 980-8574 Japan; 15 Advanced Research Center for Innovations in Next-Generation Medicine, Tohoku University, Sendai, 980-8573 Japan

**Keywords:** Aromatic amino acid metabolism, Aryl hydrocarbon receptor, Corn germ meal, Ex vivo fermentation, Microbial metabolites, Sustainable feed ingredients, Swine nutrition

## Abstract

**Background:**

Food by-products, such as corn germ meal from starch processing, are increasingly used as sustainable feed supplements, reducing competition between food and feed and supporting the valorisation of food waste. However, their effects on gut microbial metabolism and host health remain unclear. This study aimed to determine how corn germ meal fermentation influences microbial community structure and metabolite production using an ex vivo pig faecal culture system.

**Results:**

Corn germ meal supplementation significantly altered the microbial composition, increasing diversity and enriching fibre-degrading Prevotellaceae, a key bacterial family involved in complex carbohydrate metabolism. Metabolomic analysis revealed marked increases in tryptophan-derived metabolites, including indoleacrylic acid, indolepropionic acid, and indolelactic acid, which act as ligands for the aryl hydrocarbon receptor and have anti-inflammatory properties. *Prevotella*-mediated catabolite repression reduced *Escherichia coli*–derived indole formation, redirecting microbial tryptophan metabolism toward the production of these bioactive compounds. Microbial and metabolic responses differed among farms, reflecting farm-specific microbiome structures.

**Conclusions:**

Corn germ meal supplementation reshapes gut microbial communities, enhances metabolic activity, and promotes the generation of bioactive tryptophan metabolites with potential immunomodulatory effects. These findings highlight the value of corn by-products as dietary fibres that can drive beneficial microbial cross-feeding and influence host intestinal homeostasis. Although demonstrated in an ex vivo setting, this study provides a mechanistic basis and preclinical evidence for future in vivo studies, supporting the sustainable utilisation of food industry by-products to improve gut health and resource efficiency in livestock production.

**Supplementary Information:**

The online version contains supplementary material available at 10.1186/s40104-026-01364-4.

## Background

Corn is one of the most essential and widely cultivated crops worldwide, serving as a staple food, a primary livestock feed, and a critical resource for bioethanol production [1–3]. Over 80% of global corn production is allocated to livestock feed and bioethanol. The demand for corn may further increase with the rise in global population [4]. However, large-scale corn production for livestock feed and bioethanol poses significant environmental costs [5–8]. Furthermore, climate change-related shifts in temperature and rainfall may reduce corn yields. Therefore, optimisation of food resources for sustainable production is crucial [9–12].

Recent research has focused on maximising the utility of food by-products, particularly those generated during corn starch extraction [13]. Starch granules are primarily concentrated in the endosperm of corn kernels, which, on a dry basis, consists of approximately 80% endosperm, 10% germ, and 5% pericarp [13]. Non-starch components, including cellulose and hemicellulose, are polysaccharides abundant in corn by-products [13]. Initially valued for their lipid content and suitability for applications in edible oils and industrial energy, corn by-products are now increasingly recognised as a resource for livestock feed supplementation [14–18]. With rising demand for livestock products and growing environmental concern, incorporating corn by-products into feed could promote sustainability by reducing excessive corn production and alleviating competition between food and feed resources [19].

The gut microbiome critically metabolises dietary components, and these metabolic activities subsequently reshape the microbial community. Such microbiota-driven alterations can modulate host physiological phenotypes, highlighting the dynamic interplay among diet, microbes, and host [20–23]. The gut microbiome is essential for metabolising and utilising various compounds that cannot be digested or synthesised by mammals. For instance, the microbiome is critical for the fermentation of plant-derived polysaccharides and feeds resistant to mammalian digestive enzymes [24]. In the colon, anaerobic bacteria ferment these polysaccharides, producing short-chain fatty acids (SCFAs) such as acetate, propionate, and butyrate. This process often involves cross-feeding, where primary degraders, such as *Bacteroides*, *Prevotella*, and *Ruminococcus*, break down complex polysaccharides into simpler molecules, which are then utilised by secondary fermenters, such as *Faecalibacterium* and *Roseburia*, to produce SCFAs [25, 26]. For example, butyrate serves as the primary energy source for colonocytes and contributes to intestinal barrier integrity and inflammation regulation [23, 26].

In addition to SCFAs, the microbiome produces various small molecules and metabolites that act as signalling molecules mediating host-microbiome cross-talk [27]. For example, *N*-methyl serotonin, extracted from orange peel fibres during microbial decomposition, has physiological activity that contributes to prevent obesity [28]. Similarly, ferulic acid, primarily bound to arabinoxylan in corn fibre, is released during microbial degradation and can modulate oxidative stress and inflammatory responses in the host [29]. Therefore, microbial degradation of polysaccharides in plant-derived foods and feeds can release and transform various bioactive compounds, potentially enhancing their bioavailability.

Pigs (*Sus domestica*) are non-ruminant livestock that generate a lower environmental footprint than that generated by ruminants, partly due to reduced methane production and low nitrogen-related emissions [30]. Against a backdrop of rising global pork demand [31] and corn’s increasingly prominent role in animal feeds [31–34], the efficient utilisation of corn by-products is essential. The gut microbial composition influences pig growth [34, 35]. For example, feed supplemented with corn bran, a byproduct of starch, increases microbial diversity and promotes anti-inflammatory cytokine production in piglets [36]. Despite extensive research on microbial metabolism in humans, livestock feed research has largely focused on SCFA production [37–39], with limited exploration of other bioactive compounds derived from microbial degradation of polysaccharides.

This study aimed to investigate the microbial metabolism of corn germ (hereafter referred to as corn germ meal), a fibre-rich by-product commonly used as a livestock feed supplement [40]. We hypothesised that microbial fermentation of corn germ meal would shift the pig gut microbiome toward fibre-degrading taxa and enhance the production of tryptophan-derived metabolites, including AhR ligands, while suppressing indole formation. Through a comprehensive analysis of microbial metabolites generated during corn germ meal fermentation, we characterised the bioactive compounds produced by the microbiome and examined their potential functional interactions with the host. This study highlights the value of corn germ meal as a functional feed ingredient and provides new insights into how microbial metabolism of plant-derived polysaccharides yields bioactive metabolites with implications for livestock health and sustainable agriculture.

## Methods

### Animals and sample collection

Faecal samples were obtained from four commercial farms: the Miyagi Prefecture Livestock Experiment Station (14- and 9-week-old pigs; Miyagi Prefecture, Japan; farms A and D), a local farm in Gifu Prefecture, Japan (89-week-old pigs; farm B), and the Gifu Livestock Research Institute (Gifu Prefecture, Japan, 68-week-old pigs; farm C). For faecal tryptophan (Trp) and indole measurements, additional samples were collected from 82 piglets (3 weeks old; 3–4 d post-weaning) on farm B. Only clinically healthy pigs were selected for sampling. Health status was confirmed by farm staff based on the absence of diarrhoea, respiratory symptoms, abnormal behaviour, and reduced feed intake. All pigs had received routine vaccinations and were administered only farm-permitted antibiotics under standard management practices. Within each farm, pigs were maintained under the same farm-specific diet and husbandry conditions, ensuring consistency in environmental and nutritional backgrounds within farms while allowing the capture of farm-to-farm variation. To preserve farm-specific microbial characteristics and avoid dilution effects associated with pooled samples, fresh faecal material was collected directly from the rectum of a single representative healthy pig at each farm immediately after defecation. All samples were snap-frozen on site at −80 °C and transported to the laboratory on dry ice. Upon arrival, samples were thawed on ice, diluted to 0.5% (w/v) in PreserWell solution (MPR, Miyagi, Japan; stabiliser for anaerobes), and promptly re-frozen at −80 °C. The interval between faecal sample collection and initiation of fermentation experiments varied among samples, with storage at −80 °C for up to approximately 6 months prior to use.

### Faecal culture

Corn germ meal, a starch production by-product supplied by Nihon Shokuhin Kako Co., Ltd. (Shizuoka, Japan), was powdered using a blender and added to Swine Colon Medium (SCM) (Table S1, referred to as “No. 4 media” in Tsujikawa et al. [41]) at a final concentration of 5% (w/v), with its nutritional composition detailed in Table S2. Corn starch (Fujifilm Wako, Osaka, Japan; #193-09925) was similarly incorporated at 5% (w/v). Media were autoclaved at 121 °C for 15 min to minimise contamination. Frozen pig faecal samples were thawed on ice and diluted five-fold in SCM broth. The diluted faecal suspension was then added to SCM broth at a final concentration of 0.01% (v/v) in 10 mL. Cultures were incubated anaerobically at 37 °C for 50 h in loosely capped 15 mL Falcon tubes placed in sealed containers with an AnaeroPack catalyst (Mitsubishi Gas Chemical Co., Tokyo, Japan). Optical density (OD) was measured at 660 nm using a Miniphoto spectrophotometer (Titec, Tokyo, Japan). Before measurement, test tubes were inverted five times, allowed to settle for 5 s, and the OD_660_ of the supernatant was recorded.

Faecal samples were obtained from one pig at each of four different farms (farms A–D represent pigs from four distinct farms). Each faecal inoculum was cultured separately under two conditions: with and without corn germ meal supplementation. All cultures were performed in triplicate as technical replicates (*n* = 3 per faecal inoculum for each treatment condition). Microbial community and metabolite analyses were conducted using these replicated cultures. As the analyses in this study focused on relative differences between faecal culture conditions, uninoculated SCM controls were not included.

### 16S rRNA sequence

Genomic DNA was extracted from both faecal samples and bacterial isolates using the GenCheck DNA Extraction Kit (Type S/F; Fasmac Co., Ltd., Kanagawa, Japan) according to the manufacturer’s instructions. DNA extracted from faecal samples was then used for 16S rRNA gene sequencing to determine bacterial composition and read counts. The V3–V4 region of the bacterial 16S rRNA gene was amplified using the primers “Read1_341F” and “Read2_805R” [42]. For further details, refer to the Supplementary Materials and Methods. Alpha diversity metrics, including observed features and the Shannon index, were calculated at a rarefaction depth of 10,000 reads, ensuring that rarefaction curves plateaued across all samples. Beta diversity was assessed using Bray–Curtis dissimilarity at a sampling depth of 50,000 reads, and the results were visualised using principal coordinate analysis (PCoA). Functional metagenomic predictions were performed using PICRUSt2 [43] via the QIIME2 plugin (version 2023.2 #17; https://github.com/picrust/q2-picrust2/pull/17).

### Metabolomic analysis and faecal metabolite quantification

Metabolomic profiling was conducted using capillary electrophoresis time-of-flight mass spectrometry (CE-TOF-MS) with the Basic Scan package provided by Human Metabolome Technologies, Inc. (HMT; Yamagata, Japan).

For further details, refer to the Supplementary Materials and Methods. For statistical analysis, values below the detection limit were replaced with zero, and metabolites that were not detected in any samples were excluded from further analysis. Detected compounds were classified based on metabolic pathways in the KEGG Pathway Database (https://www.kegg.jp/pathway/map01100).

### Cell culture

Swine intestinal epithelial cells (SIECs) [44] were cultured in growth medium consisting of DMEM/F12 supplemented with 1 × insulin-transferrin-selenium (Merck/Millipore Sigma, Burlington, MA, USA), 5 ng/mL recombinant human EGF (Merck/Millipore Sigma), 10% heat-inactivated FBS, and 1% penicillin–streptomycin (Thermo Fisher Scientific). Cells were seeded into collagen-type I-C-coated flasks or plates (Cell-tight C-1, Sumitomo Bakelite, Tokyo, Japan) and maintained at 37 °C in a humidified incubator with 5% CO_2_. SIECs were used within 10 passages after thawing, and the line was routinely passaged every 5 d.

### Cell treatment

SIECs were seeded at a density of 1 × 10^4^ cells/well in a collagen type I-C-coated 24-well plate and incubated overnight in growth medium containing penicillin–streptomycin. On d 3, cells were treated with 100 μmol/L of one of the following compounds: 6-formylindolo[3,2-b] carbazole (Funakoshi, Tokyo, Japan), indoleacrylic acid (IA; Tokyo Chemical Industry [TCI], Tokyo, Japan), indole-3-propionic acid (IPA; TCI), indole-3-lactic acid (ILA; Combi Blocks, San Diego, USA), Trp (TCI), phenylalanine (Phe; TCI), tyrosine (Tyr; TCI), kynurenic acid (Kyn, TCI), L-DOPA (TCI), or phenyllactic acid (PLA, TCI), or with the vehicle control (DMSO). The metabolite concentration was set at 100 μmol/L based on preliminary tests showing sufficient responsiveness of SIECs. Cells were harvested for further analysis after 6 h. To investigate the role of AhR signalling, CH-223191, an AhR antagonist, was applied. For this, SIECs cultured for 5 d were pretreated with CH-223191 (10 µmol/L; Sigma-Aldrich, USA) or vehicle (DMSO) for 2 h, followed by treatment with IA, IPA, or ILA (100 µmol/L). For inflammatory stimulation, lipopolysaccharide (LPS) from *E. coli* O55:B5 (L6529; Sigma-Aldrich) or vehicle (PBS) was added to SIECs that were incubated for 5 d following pretreatment with IA, IPA, or ILA (100 µmol/L) or vehicle (DMSO) for 48 h. Cytotoxicity at the applied metabolite concentrations and exposure times was assessed using a Cell Counting Kit-8 (Dojindo Laboratories, Kumamoto, Japan), and no reduction in SIEC viability was observed (data not shown).

### Gene expression analysis for SIECs

#### RNA extraction and cDNA synthesis

Total RNA was extracted from SIECs using the TRIzol reagent (Invitrogen, Carlsbad, CA, USA) following the manufacturer’s instructions. The RNA concentration and purity were evaluated using a NanoDrop^®^ ND-1000 spectrophotometer. To synthesise cDNA, 1,000 ng of total RNA was reverse-transcribed using the PrimeScript RT reagent Kit (Takara Bio, Shiga, Japan) following the manufacturer’s protocol. The synthesised cDNA was diluted sixfold with nuclease-free water and stored at −30 °C.

#### Quantitative PCR (qPCR)

For qPCR, 2.5 µL of cDNA, 1 µmol/L of each primer (Table S3), and TB Green Premix Ex Taq II (Takara Bio) were used in a CFX Connect Real-Time PCR System (Bio-Rad). The amplification conditions included an initial denaturation at 95 °C for 30 s, followed by 40 cycles at 95 °C for 5 s and 60 °C for 30 s. β-Actin was used as the reference gene because its expression has been shown to remain stable in SIECs under inflammatory stimulation [41], and preliminary assessments confirmed minimal variation across treatments.

### Bacteria and culture conditions

*Prevotella copri* JCM13467 (*Segatella copri*) was obtained from the RIKEN BioResource Centre, whereas *Escherichia coli* ME9062 (WT) and *E. coli* JW3686-KC (*ΔtnaA*, tryptophanase) were obtained from the Keio Collection (NBRP, Mishima, Shizuoka). *P. copri* was cultured on PYG medium (DSM 104) under anaerobic conditions (85% N_2_, 10% CO_2_, 5% H_2_) using an anaerobic chamber (COY Laboratory Products, Grass Lake, MI, USA) at 37 °C. Wild-type (WT) *E. coli* was cultured in LB broth, whereas *E. coli* (*ΔtnaA*) was cultured on LB broth supplemented with 0.1% glucose and 100 µg/mL kanamycin, with continuous shaking (180 r/min) at 37 °C. Prior to experiments, all strains were streaked on their respective agar media (PYG agar for *P. copri*; LB agar or for *E. coli*), and single colonies were isolated to confirm culture purity. Thereafter, 20% (w/v) glycerol stocks were prepared and stored at −80 °C.

### Co-culture of *P. copri* and *E. coli*

*E. coli* WT and *ΔtnaA* strains were pre-cultured in LB broth, with *E. coli ΔtnaA* grown in LB broth supplemented with 0.1% glucose and 100 µg/mL kanamycin. *P. copri* was cultured in PYG broth. All strains were cultured until early exponential phase to ensure consistency across experiments, and the cultures were then diluted in LB broth (*E. coli* WT and *ΔtnaA*) or PYG broth (*P. copri*) to achieve an OD_600_ of approximately 1.2. Following dilution, 1% (v/v) *E. coli* WT or *ΔtnaA* and 3% (v/v) *P. copri* were inoculated into 4 mL of SCM broth. The cultures were incubated at 37 °C in an anaerobic chamber, and bacterial samples were collected at 20 and 50 h. Samples collected at 20 h were used for mRNA expression analysis, whereas those collected at 50 h were subjected to quantification of Trp derivatives via liquid chromatography-tandem mass spectrometry (LC–MS/MS).

### Quantification of aromatic amino acids (AAA) and its derivatives using LC–MS/MS

#### Sample preparation

Supernatant from ex vivo faecal samples and bacterial culture media were mixed with methanol at a 1:1 ratio, vortexed vigorously, and incubated on ice for 1 h to facilitate protein precipitation. The mixtures were then centrifuged at 10,000 × *g* and 4 °C for 10 min. The resulting supernatants were filtered through a 0.2-µm filter (NACALAI TESQUE, Kyoto, Japan) and centrifuged again at 10,000 × *g* and 4 °C for 10 min. The filtrates were further processed using a 3 kDa molecular weight cutoff filter (APRO SCIENCE, Tokushima, Japan), and centrifugation was performed until sufficient filtrate was obtained. The collected samples were stored at −30 °C.

Faecal samples, excluding those from farms A and D, were suspended in water (1 mg of faeces per 20 µL of water).

Faecal samples from farms A and D were suspended in a more diluted solution, with a 1:19 ratio (1 µL faecal suspension to 19 µL water). These suspensions were then mixed with 100% methanol in a 1:1 ratio, vortexed vigorously, and incubated on ice for 1 h to facilitate protein precipitation. After incubation, the samples were centrifuged at 10,000 × *g* for 10 min at 4 °C. The resulting supernatants were filtered through a 0.2-µm filter and centrifuged again under the same conditions. To further concentrate the metabolites, the filtrates were processed using a 3 kDa molecular weight cutoff filter, with centrifugation performed until enough filtrate was collected. The final samples were stored at −30 °C.

#### LC–MS/MS conditions

LC–MS/MS was performed using a QTRAP6500 quadrupole linear ion trap hybrid tandem mass spectrometer (SCIEX, Framingham, MA, USA) with an ESI probe attached to the ion source, coupled with an ultra-high-performance liquid chromatograph Nexera (Shimadzu, Kyoto, Japan). Measurements were conducted in negative ion mode. Analyst 1.6.2 (SCIEX) and MultiQuant version 2.1.1 (SCIEX) software were used for data analysis and peak area integration, respectively. Statistical analysis was performed using JMP Pro version 17.1 software (SAS Institute Inc., Cary, NC, USA). A consisted of formic acid and 10 mmol/L ammonium formate solution in water (0.1:100 dilution) and B consisted of methanol, respectively, in the gradient flow mode. The flow rate was adjusted to 0.3 mL/min. An InnertSustain AQ-C18 (2.1 mm i.d. × 150 mm, GL-sciences, Tokyo, Japan) was used as the column, and the column temperature was set at 40 °C. The composition of mobile phase B increased from 5% to 95% from 0 to 6 min, from 6 to 8 min at 95% and from 8 to 10 min equilibrating to initial conditions. Prepared samples were mixed with ISs at a ratio of 1:1, and a 3-µL volume was injected for analysis.

For the quantification of Trp metabolites excluding indole, a 16-point calibration curve was prepared using 50% methanol (methanol:water, 1:1) as the solvent for the prepared samples. The concentrations for the calibration curve ranged from 0.1 to 40 µmol/L with the following values: 40, 30, 20, 10, 8, 5, 4, 3, and 2 µmol/L. The LC–MS/MS source conditions were as follows: gas 1 at 60 psi, gas 2 at 70 psi, collision gas at 11 psi, curtain gas at 45 psi, temperature at 700 °C, and IonSpray voltage at −4,500 V. The MRM algorithm used for analysis is indicated in Table S4. For IS, 10 µmol/L indoleacetic acid-d_5_ was used for indoleacetic acid, and 10 µmol/L Trp-d_5_ was used for the other Trp derivative.

For the quantification of indole, a 16-point standard curve was prepared using 50% methanol as the solvent for the prepared samples. The concentrations for the standard curve ranged from 0.4 to 50 µmol/L, with the following values: 50, 40, 30, 25, 20, 15, 10, 8, 5, 4, 3, and 2 µmol/L. The LC–MS/MS source conditions were set as follows: gas 1 at 30 psi, gas 2 at 40 psi, collision gas at 12 psi, curtain gas at 10 psi, temperature at 700 °C, and IonSpray voltage at 5,500 V. The MRM algorithm used for analysis is indicated in Table S5. For IS, 10 µmol/L indoleacetic acid-d_5_ was used.

### Statistical analysis

Statistical analyses were performed using GraphPad Prism (version 10.2.0; GraphPad Software, Boston, MA, USA), unless otherwise specified. Comparisons between two groups were conducted using unpaired or paired two-tailed Student’s* t*-tests, as appropriate. For comparisons among multiple groups, one-way analysis of variance (ANOVA) followed by Tukey’s or Dunnett’s post hoc test was applied. Non-parametric data were analysed using the Mann–Whitney *U* test or Wilcoxon signed-rank test. Correlations were assessed using Spearman’s rank correlation coefficient. Microbiome beta-diversity differences were evaluated by PERMANOVA, as specified in the corresponding figure legends. Where applicable, *P* values were adjusted for multiple comparisons using the Benjamini–Hochberg false discovery rate (FDR). Statistical significance was defined as *P* < 0.05. The specific statistical tests used for each dataset are indicated in the corresponding figure legends.

## Results

### Impact of corn germ meal on microbial composition and metabolite profiles

**Fig. 1:**
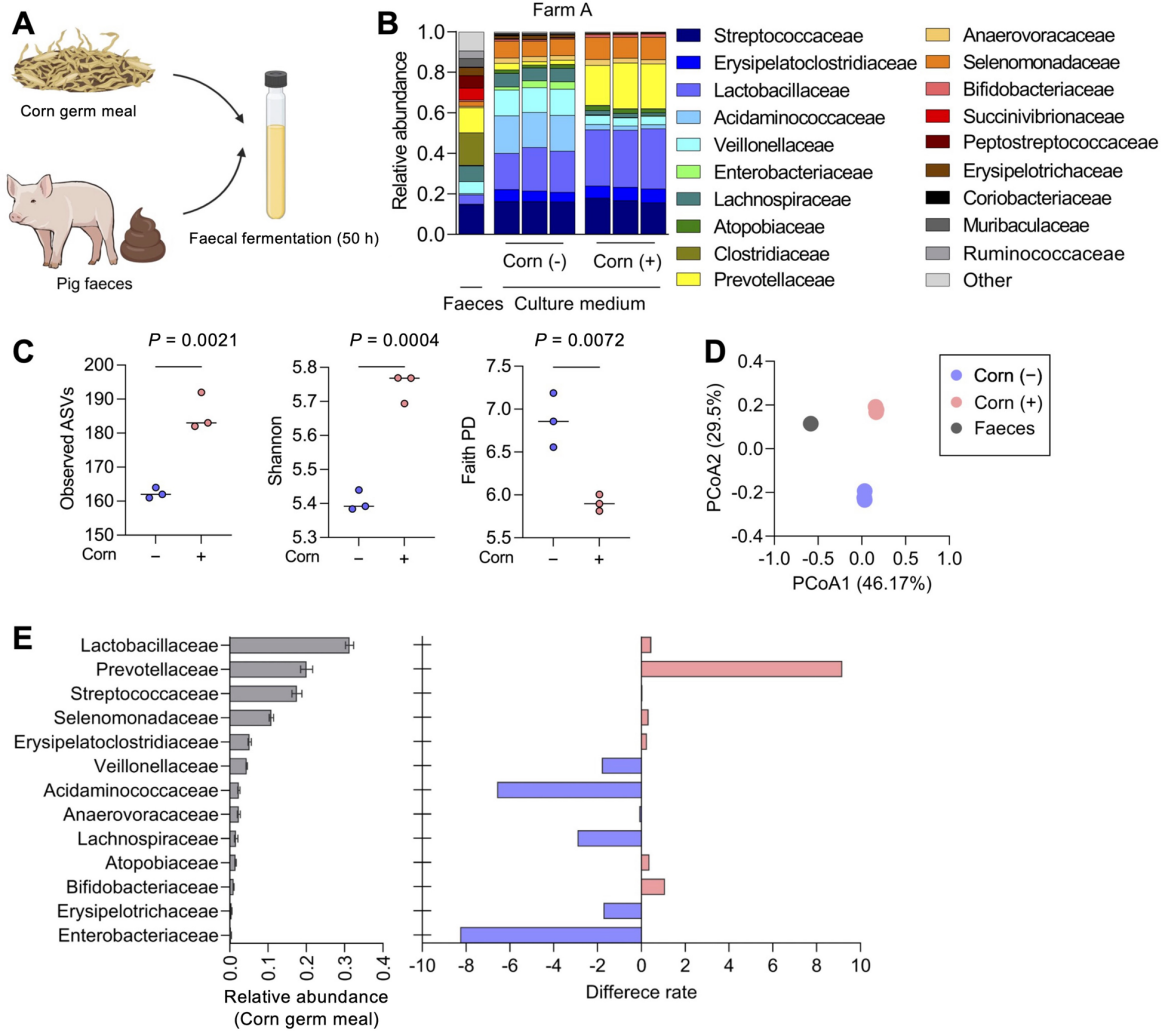
Impact of corn germ meal supplementation on pig microbial composition. **A** Experimental design for the ex vivo fermentation assay using pig faecal cultures with or without 5% (w/v) corn germ meal supplementation (created with Biorender.com). **B** Relative abundance of bacterial families detected in original faecal samples and their corresponding cultures after 50 h of fermentation in the presence or absence of corn germ meal. Each bar represents the microbial composition derived from the faeces of an individual pig (Farm A). Cultures were performed in technical triplicate for each treatment condition (*n* = 3 per faecal inoculum). **C** Alpha diversity indices (Observed ASVs, Faith’s PD and Shannon index) of microbial communities in control and corn germ meal-supplemented cultures at 50 h fermentation. Each data point corresponds to a technical replicate (*n* = 3 per faecal inoculum), with horizontal bars indicating the median. Statistical significance was determined using the Mann–Whitney *U* test. **D** Principal coordinate analysis (PCoA) based on Bray–Curtis dissimilarity illustrating shifts in microbial community structure induced by corn germ meal supplementation (Table S8). **E** (Left) Relative abundance of major bacterial families (> 1% of total community) with and without corn germ meal supplementation (mean ± SD, *n* = 3 per treatment). (Right) Horizontal bars indicate the difference in mean relative abundance (percentage points) between cultures with and without corn germ meal. Blue and red indicate decreased and increased abundances, respectively

To examine the effects of corn germ meal supplementation on the gut microbial composition of pigs, we utilised an ex vivo pig faecal culture model [41] to comprehensively analyse microbial community composition and metabolic activity. Anaerobic fermentation was conducted by inoculating pig faeces (farm A) into SCM broth [41] with 5% (w/v) corn germ meal. OD values increased continuously for 50 h (Fig. S1) with slightly lower levels in supplemented cultures, suggesting altered microbial activity or substrate utilisation. Based on this growth pattern, 16S rRNA sequencing was performed using samples collected at 25 h and 50 h, corresponding to early and late stages of exponential growth, respectively. Sequencing data were analysed using the QIIME2 pipeline (Fig. S2A). Alpha diversity indices consistently indicated higher diversity at 50 h than at 25 h (Fig. S2B). The microbial community retained its compositional complexity at 50 h (Fig. S2A and B); therefore, 50 h was used as the optimal time point for further analyses. Evaluation of the effect of corn germ meal supplementation on microbial composition revealed altered microbial family-level profiles (Fig. 1B) and significantly increased alpha diversity (Fig. 1C) following corn germ meal addition. Beta diversity analyses, including Bray–Curtis and weighted UniFrac PCoA, confirmed corn germ meal–induced distinct shifts in microbial community structure compared with controls (Fig. 1D and Fig. S3A).

Thirteen bacterial families with > 1% mean relative abundance in the presence of corn germ meals were ranked accordingly. Figure 1E presents the difference in relative abundance (absolute difference in percentage points) between cultures with and without corn germ meal. Corn germ meal supplementation resulted in significant enrichment of Lactobacillaceae, Prevotellaceae, Selenomonadaceae, Atopobiaceae, and Bifidobacteriaceae, with Prevotellaceae showing the highest relative increase. The relative abundances of Lactobacillaceae, Selenomonadaceae, Atopobiaceae, and Bifidobacteriaceae also increased markedly (Fig. S3B). Conversely, Veillonellaceae, Acidaminococcaceae, and Lachnospiraceae exhibited significant reductions following corn germ meal supplementation (Fig. 1E and Fig. S3B). Therefore, corn germ meal supplementation substantially altered the microbial community, enhancing the diversity and abundance of bacterial taxa capable of metabolising the components of corn germ meal.

### Corn germ meal enhances microbial metabolism of AAAs

**Fig. 2:**
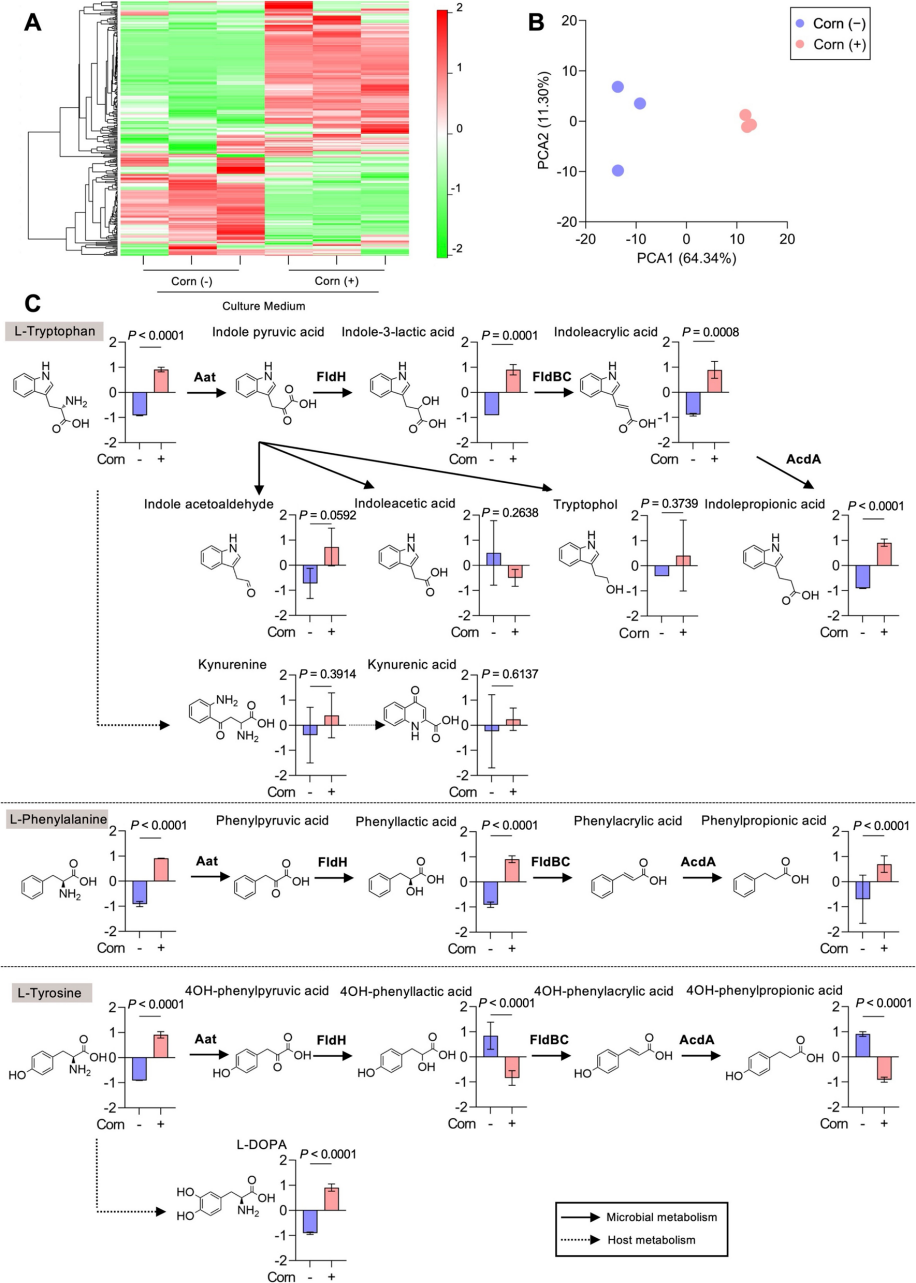
Corn germ meal enhances microbial metabolism of aromatic compounds. **A** Metabolomic analysis of ex vivo faecal fermentation cultures using capillary electrophoresis time-of-flight mass spectrometry (CE-TOF-MS) identified a total of 280 candidate metabolites. Hierarchical clustering shows metabolite distribution patterns. The heatmap illustrates relative abundance changes of faecal metabolites following corn germ meal supplementation. Each treatment was performed in technical triplicate (*n* = 3 per faecal inoculum) using samples from a single pig from Farm A. Red and green indicate high and low metabolite levels, respectively. A total of 61 and 35 metabolites were significantly increased or decreased, respectively, based on unpaired *t*-tests (*P* < 0.05; see Table S6). **B** Principal coordinate analysis (PCoA) plot based on Bray–Curtis dissimilarity illustrates shifts in the faecal metabolomic profile following corn germ meal supplementation (*n* = 3 per treatment). **C** Schematic of microbial or mammalian aromatic amino acid (AAA) metabolism in the gut, highlighting the tryptophan (Trp), phenylalanine (Phe), and tyrosine (Tyr) pathways and their derivatives detected in this study. Metabolites are ordered according to the Stickland fermentation pathway by *Clostridium sporogenes* [45, 46]. Stickland fermentation of AAA generates arylacetates, such as indoleacetic acid (IAA) in the oxidative pathway. In the reductive pathway, aryllactates (i.e., indolelactic acid, phenyllactic acid, and 4-hydroxy phenyllactic acid) are generated by phenyllactate dehydrogenase from aryl pyruvates. Aryllactates are converted to arylacrylates (i.e., indoleacrylic acid, phenyllactic acid, and 4-hydroxy phenylacrylic acid) by phenyllactate dehydratase. Furthermore, arylacrylates are converted to arylpropionates (such as indolepropionic acid, phenylpropionic acid, and 4-hydroxy phenylpropionic acid) by acyl-CoA dehydrogenase. Kynurenine and kynurenic acid (KyA) are produced through the kynurenine pathway by host metabolism [47]. L-DOPA is produced from Tyr by tyrosine hydroxylase [48]. Tryptophol is produced from indolepyruvic acid by *Candida albicans* [49]. Solid or dotted lines indicate microbial or host metabolism. Metabolite levels are shown as the mean ± SD (*n* = 3 per condition). *P*-value is determined using an unpaired *t*-test

Corn germ meal supplementation induced substantial changes in the metabolomic profile. A total of 280 compounds were detected, including 197 and 83 in cation and anion modes, respectively. Among these, 61 and 35 compounds were significantly increased and decreased, respectively, following supplementation (Fig. 2A, B; see full statistical results in Table S6). The detected compounds were then classified using the KEGG pathway database to better understand the characteristics of the altered metabolites. KEGG pathway classification revealed a significant enrichment of compounds involved in amino acid metabolism, suggesting that the observed metabolic changes were largely driven by amino acid-related metabolites (Fig. S4A). Notably, most individual amino acid levels, particularly AAAs, were increased following supplementation, except for aspartic acid (Fig. S4B). However, corn germ meal supplementation modestly altered SCFA levels, with a significant increase in acetate and a decrease in butyrate observed (Fig. S4C). Considering the pronounced increase in amino acid-related metabolites, subsequent analyses focused on AAAs and their derivatives.

Figure 2C illustrates the AAAs—Trp, Phe, and Tyr—and their related metabolites detected in faecal cultures, alongside key AAA-metabolising enzymes present either in bacteria, predominantly *Clostridium sporogenes*, or in mammalian cells [45, 47]. Trp metabolism was markedly enhanced in response to corn germ meal supplementation, as evidenced by significant increases in Trp-derived metabolites, including ILA, IA, and IPA (Fig. 2C). Notably, Kyn and kynurenic acid (KyA), products of mammalian-mediated Trp metabolism, were also slightly elevated following supplementation. Regarding Phe, corn germ meal supplementation increased PLA and phenylpropionic acid (PPA) levels, whereas the intermediary metabolites phenylacrylic acid and phenylpyruvic acid were not detected (Fig. 2C). Similarly, Tyr metabolism was influenced by corn germ meal supplementation, as indicated by increased levels of Tyr-derived metabolites such as L-DOPA. However, the levels of 4-hydroxyphenyllactic acid and 4-hydroxyphenylpropionic acid decreased (Fig. 2C). Combined with the microbial analysis, these results suggest that IPA and PPA may be produced through as-yet-unidentified pathways, as *C. sporogenes* and *Peptostreptococcus anaerobius*, which possesses acyl-CoA dehydrogenase, were not detected (Table S7) [45]. Notably, AAA metabolism is significantly enhanced by corn germ meal supplementation, reflecting increased metabolic activity of the microbiome.

### Corn germ meal–derived microbial metabolites activate AhR

**Fig. 3:**
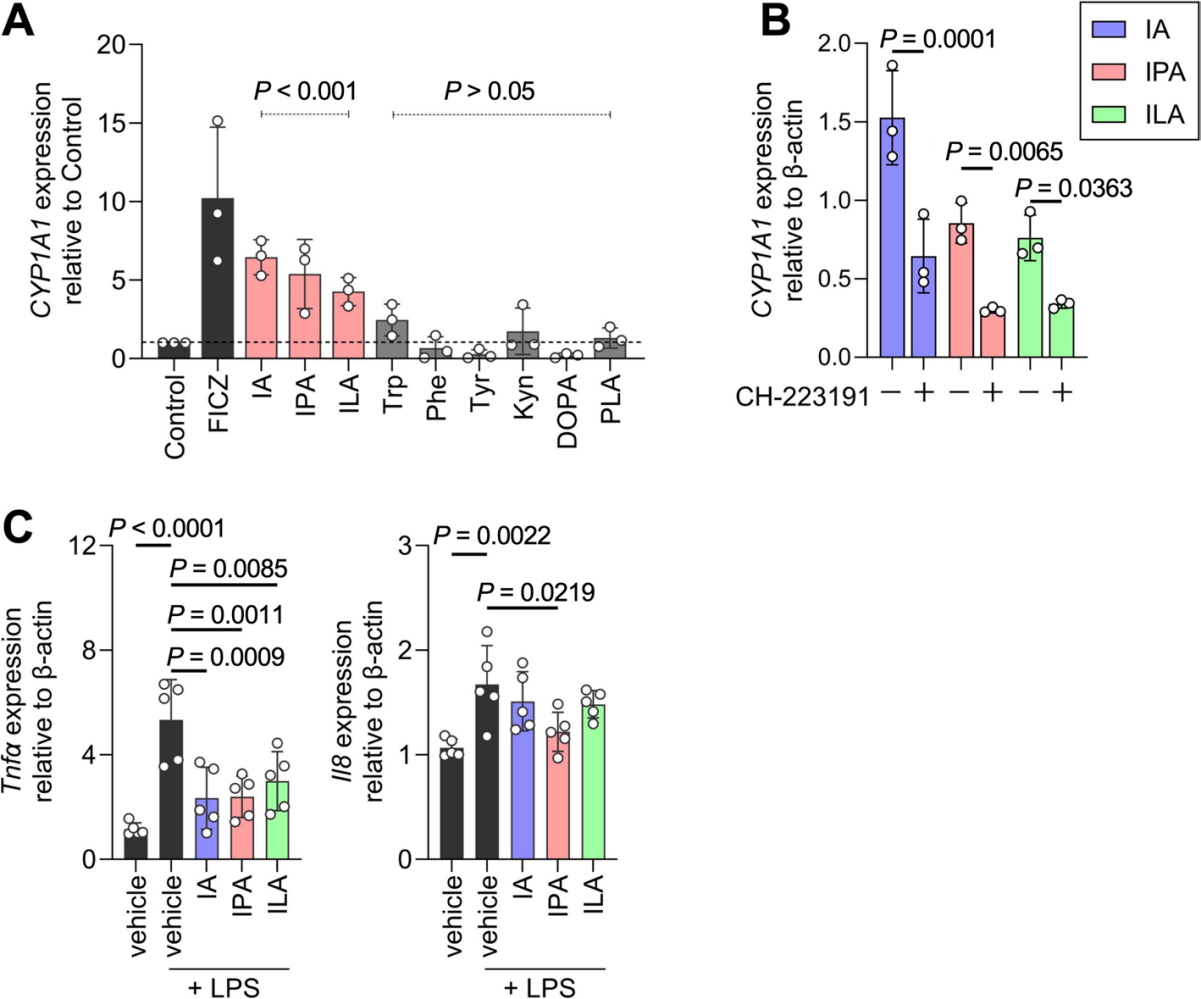
Corn germ meal-derived microbial metabolites activate the aryl hydrocarbon receptor (AhR). **A** RT-qPCR analysis of CYP1A1 expression in swine intestinal epithelial cells (SIECs) stimulated with aromatic metabolites (100 µmol/L) for 6 h detected in faecal cultures (mean ± SD, *n* = 3 per condition). **B** CYP1A1 expression following pretreatment with AhR antagonist (CH-223191; 10 µmol/L) for 2 h prior to AhR ligand stimulation (mean ± SD, *n* = 3 per condition). **C** Modulation of inflammatory responses in SIECs: *Tnfα* and *Il8* expression after LPS (1 µg/mL) stimulation followed by AhR ligands (100 µmol/L) or vehicle (DMSO) stimulation for 48 h (mean ± SD, *n* = 5 per condition). Each dot represents an independent biological replicate. *P* < 0.05 was considered significant and determined using one-way ANOVA followed by Bonferroni’s test (**A**), two-way ANOVA followed by Bonferroni’s test (**B**), or one-way ANOVA followed by Dunnett’s test (**C**)

Based on the CE-TOF-MS results, we hypothesised that bacterial metabolites elevated by corn germ meal supplementation could activate AhR signalling pathways in pigs (Fig. 2C). Therefore, we assessed whether aromatic compounds detected using CE-TOF-MS could activate AhR in SIECs. SIECs were stimulated with several aromatic compounds increased by corn germ meal supplementation (Fig. 3), and the expression of *CYP1A1*, a well-established downstream target of AhR, was quantified via RT-qPCR. AhR activation induces *CYP1A1* expression, thereby mediating ligand degradation [50]. Notably, *CYP1A1* expression in SIECs was significantly induced by IA, IPA, and ILA (Fig. 3A). Furthermore, pretreatment of SIECs with CH-223191, an AhR antagonist, significantly suppressed *CYP1A1* expression following IA, IPA, and ILA stimulation, confirming that their effects were AhR-mediated (Fig. 5B). These results identify IA, IPA, and ILA as potential AhR ligands in pigs.

Interestingly, although KyA is a potent endogenous AhR ligand in humans [51, 52], it failed to induce *CYP1A1* expression in our study (Fig. 3A). To examine whether structural differences in AhR could account for the observed discrepancy between pigs and humans, we performed structural modelling of AhR using AlphaFoldDB and compared the predicted porcine AhR structure with available experimental and predicted AhR structures from structural databases. The predicted model revealed subtle differences in the ligand-binding site of porcine AhR compared with its human counterpart, which may affect ligand recognition and binding specificity (Fig. S5). These findings suggest that microbial metabolites enhanced by corn germ meal supplementation exhibit species-specific bioactivity through AhR signalling pathways. Moreover, LPS, which induces inflammation in epithelial cells, was added to SIECs after treatment with AhR ligands. AhR regulates intestinal immunity through its interaction with nuclear factor-κB and other immune modulators [50]. As expected, LPS induced mRNA expression of the inflammatory cytokine *TNFα* and the chemokine *IL-8*, which was downregulated by stimulation of AhR ligands (Fig. 5C), suggesting that specific metabolites present following corn germ meal supplementation contribute to modulation of the host inflammatory response.

### Impact of corn germ meal supplementation on faecal microbial community and metabolite profiles in pigs from different farms

**Fig. 4:**
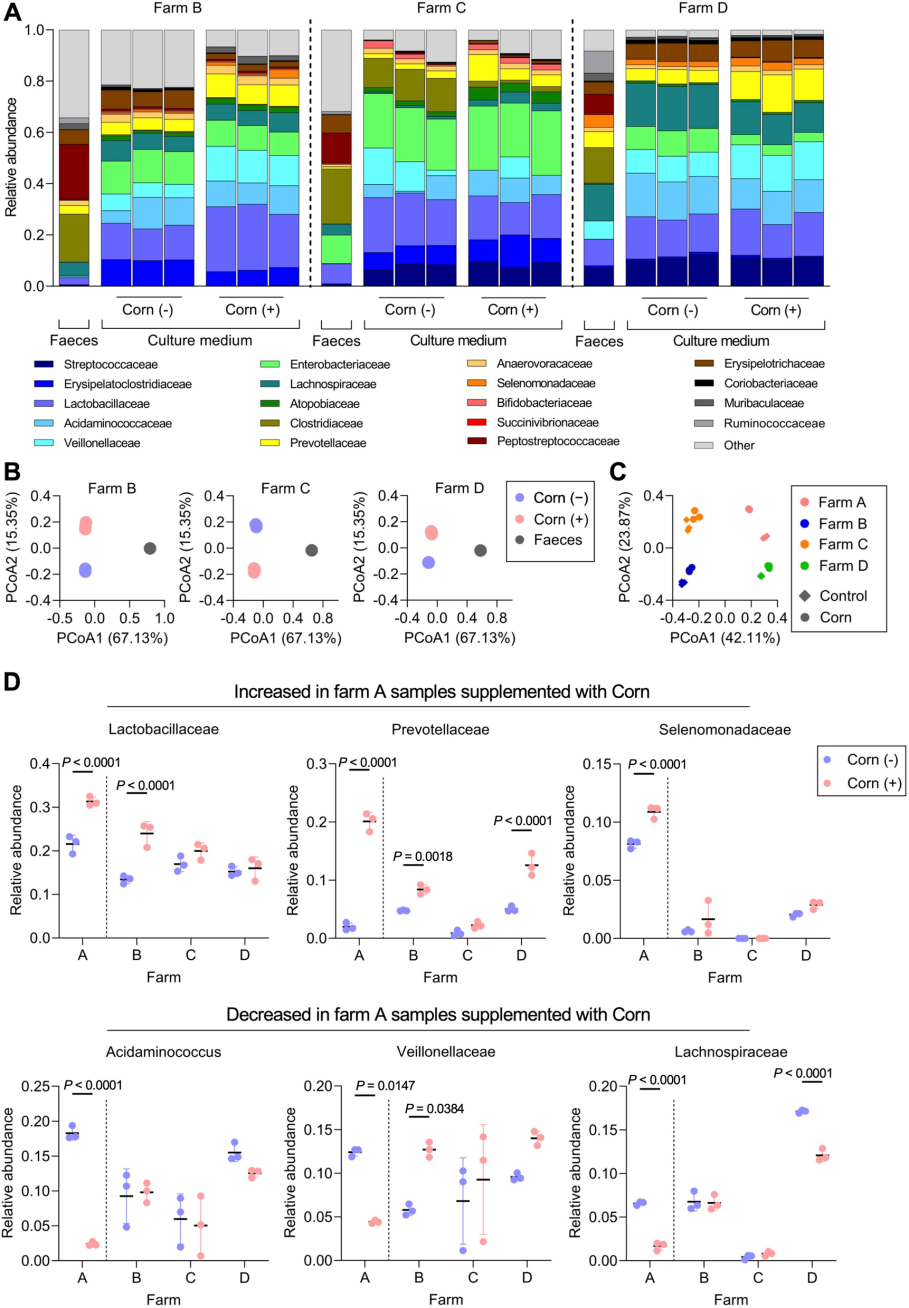
Corn germ meal supplementation differentially alters microbial composition across farms. **A** Relative abundance of major bacterial families (> 90% of total community) following corn germ meal supplementation across different farms (B–D). **B** PCoA plots of microbial community structure based on Bray–Curtis distance across three farms (B–D) represent a shift following corn germ meal supplementation (*n* = 3 per treatment) (Table S9). **C** PCoA plots show greater differences among farms (A–D) than those associated with corn germ meal supplementation in microbial composition. **D** Relative abundance of 6 bacterial families across 4 farms (A–D) is shown, each accounting for ≥ 5% of the community and showing significant changes during Farm A fermentation (Fig. S3B), are presented across four farms. Prevotellaceae abundance showed consistent increases in response to corn germ meal (mean ± SD, *n* = 3 per treatment).* P* < 0.05 using two-way ANOVA followed by Bonferroni’s test. Each treatment was conducted using faecal inoculum from a single pig per farm, with technical triplicates (*n* = 3 per faecal inoculum)

To evaluate whether corn germ meal supplementation consistently induces variations in microbial composition and metabolites in the faecal microbiome of pigs raised under different environmental conditions, we analysed faecal samples from three additional farms (designated as farms B–D). Microbial compositions differed among the original faecal samples from these farms (Fig. S6A). These samples were anaerobically fermented for 50 h, and microbial composition was confirmed via 16S rRNA sequencing. Corn germ meal significantly altered microbial composition across all farms (Fig. 4A, B). However, inter-farm differences in microbial composition were more pronounced than the variations induced by corn germ meal supplementation (Fig. 4C). The effect of corn germ meal supplementation on microbial composition differed among farms. Alpha diversity remained unchanged in samples from farms B–D, whereas a significant increase was observed in samples from farm A (Fig. S6B). We focused on major bacterial families comprising at least 5% of the microbial community and exhibiting significant increases or decreases in farm A samples during fermentation (Fig. 1E and Fig. S3B). Although most bacterial families displayed distinct change patterns across farms, Prevotellaceae consistently followed a similar trajectory, with increased relative abundance in the presence of corn germ meal (Fig. 4D). *Prevotella* spp. utilise fibre and are particularly efficient at metabolising arabinoxylan, a key component of corn [53]. Based on the consistent increase in Prevotellaceae across farms, *Prevotella* abundance was further quantified by qPCR. In farm A, *Prevotella* abundance was significantly higher in the presence of corn germ meal compared with the other farms. A similar increasing trend was noted across all farms (Fig. S7).

**Fig. 5:**
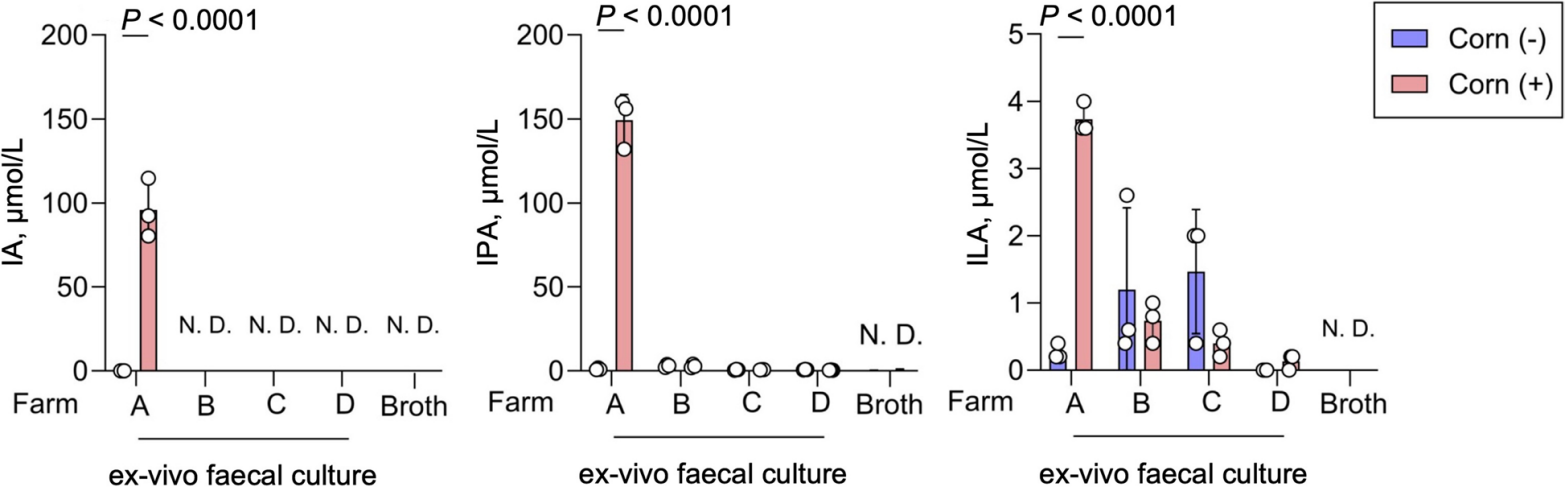
Farm-specific variation in microbial production of aryl hydrocarbon receptor (AhR) ligands. LC–MS/MS analysis quantifying the concentrations of indoleacrylic acid (IA), indolepropionic acid (IPA), and indole-3-lactic acid (ILA) in faecal culture supernatants from different farms (farms A–D). Each dot represents an independent biological replicate. Data are presented as the mean ± SD (*n* = 3 per faecal inoculum). Statistical significance was determined using two-way ANOVA followed by Bonferroni’s post-hoc test (*P* < 0.05)

Next, we quantified IA, IPA, and ILA concentrations—candidate AhR ligands identified in the SIEC cell assay—using LC–MS/MS. We assessed whether the corn germ meal–induced increases observed in farm A were consistently replicated in samples from the other farms. In samples from farm A, IA and IPA concentrations exceeded 100 µmol/L in the presence of corn germ meal (Fig. 5), reaching concentrations sufficient to activate AhR. However, in samples from farms B–D, these AhR ligands were either present at very low concentrations or undetectable (Fig. 5). The differences in AhR ligand production across farms (Fig. S8) indicate that farm-specific microbial and metabolic responses modulate the production of bioactive metabolites, highlighting the complex interactions between dietary components, inter-farm microbial community differences, and resulting metabolic outputs.

### Corn germ meal modulates indole and Trp metabolism through *Prevotella*-driven fibre degradation

**Fig. 6:**
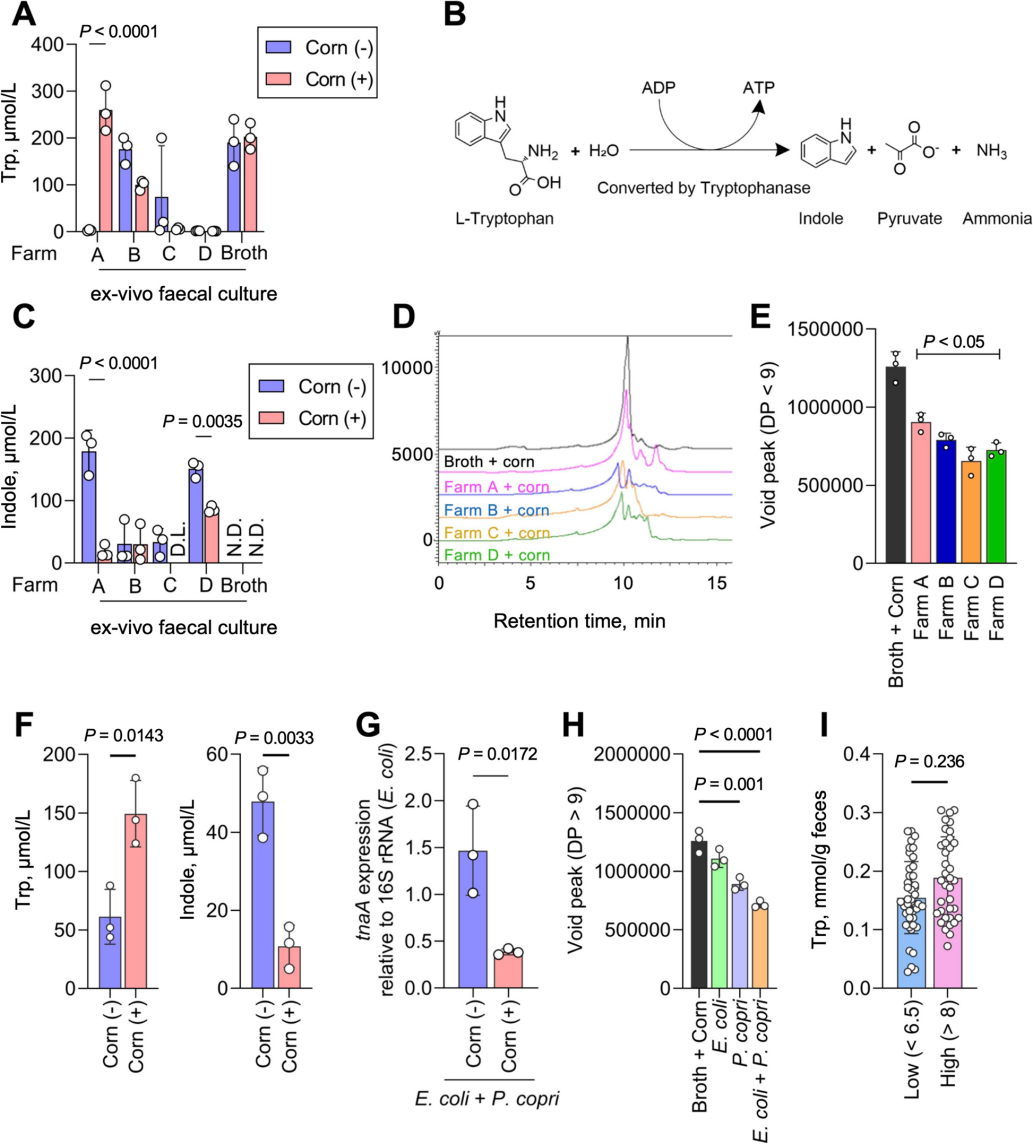
*Prevotella*-driven fibre degradation modulates tryptophan metabolism and indole production. **A** Tryptophan concentration in faecal cultures across farms (mean ± SD, *n* = 3 per condition). *P* < 0.05 using two-way ANOVA followed by Bonferroni’s test. **B** Illustration of tryptophan catabolism via tryptophanase, where bacteria release ATP and produce pyruvate and ammonia. **C** LC–MS/MS quantification of indole levels in faecal cultures from farms A and D, showing reduced indole levels in the presence of corn germ meal (mean ± SD, *n* = 3 per condition). *P* < 0.05 using two-way ANOVA followed by Bonferroni’s test. **D** Gel filtration chromatography analysis of faecal culture supernatants from farms A–D reveals the degradation of carbohydrates derived from corn germ meal. The main peak corresponds to polysaccharides, whereas minor peaks indicate degradation products formed during microbial fermentation. **E** Void peak of carbohydrates (DP > 9) in faecal culture medium (farms A–D) in the presence of corn germ meal, analysed through HPLC (mean ± SD, *n* = 3 per condition). *P* < 0.05 using two-way ANOVA followed by Bonferroni’s test. **F** Indole and Tryptophan concentrations in co-culture experiments with *Prevotella copri* and *E. coli* in the presence or absence of corn germ meal (mean ± SD, *n* = 3 per condition). *P* < 0.05 using Multipl *t*-test. **G** RT-qPCR analysis of *E. coli TnaA* expression during co-culture with *P. copri* and *E. coli* (mean ± SD, *n* = 3 per condition) for 20 h. *P* < 0.05 using an unpaired *t*-test. **H** Void peak of carbohydrates (DP > 9) in co-culture medium consisting of *P. copri* and *E. coli* in the presence of corn germ meal via HPLC analysis (mean ± SD, *n* = 3 per condition). *P* < 0.05 using two-way ANOVA followed by Bonferroni’s test. **A–H** Each dot represents an independent biological replicate. **I** Concentrations of tryptophan and indole in faeces from 82 pigs were determined using LC–MS/MS. Pigs were classified into two groups based on body weight: low (< 6.5 kg; *n* = 45) and high (> 8 kg; *n* = 37) (mean ± SD, *n* = 44 in the low group and *n* = 36 in the high group). Undetectable samples and outliers, determined using Grubbs (Alpha = 0.05), were excluded from the analysis. *P* < 0.05 using an unpaired *t*-test

To investigate the observed differences in Trp metabolism across different farms, we first quantified Trp concentrations in the fermentation medium. Trp concentrations were highest in samples from farm A, where a diverse array of Trp metabolites was uniquely detected in faeces (Fig. 5). The sustained elevation of Trp in farm A compared with other farms (Fig. 6A) was consistent with the release of Trp from proteins or peptides present in the medium or derived from corn germ meal (Table S2). Given the elevated Trp concentrations observed in farm A, the abundance of *Prevotella* spp., a genus reported to possess strong peptidase activity [54], was quantified. *Prevotella* abundance was higher in samples supplemented with corn germ meal, coinciding with increased Trp concentrations (Figs. S7A and S9). Indole, a tryptophan catabolite produced by various bacteria that yields ATP along with pyruvate and ammonia during its formation (Fig. 6B) [55, 56], was quantified across all farm samples using LC–MS/MS. In samples from farms A and D, the presence of corn germ meal was associated with a significant decrease in indole concentration compared with samples without corn germ meals (Fig. 6C). Analysis of the carbohydrate composition in the faecal culture medium using high-performance liquid chromatography (HPLC) revealed a shift of the peak corresponding to polysaccharides (degree of polymerisation [DP] of > 9) to another peak in the faecal culture medium (Fig. 6D, E), indicating enhanced microbial degradation of complex carbohydrates. To further examine whether *P. copri* and corn germ meal contribute to reduced indole production, we conducted a co-culture experiment with *P. copri* and *E. coli*. After 50 h of fermentation in SCM broth, the presence of both *P. copri* and corn germ meal significantly reduced indole concentrations, resulting in increased availability of Trp (Fig. 6F and Fig. S10). The underlying mechanism was explored by analysing *TnaA* (b3708) expression, which encodes tryptophanase, using RT-qPCR. As expected, *TnaA* expression was significantly downregulated in the presence of both *P. copri* and corn germ meal (Fig. 6G). Moreover, detection of polysaccharides (DP > 9) was significantly lower in cultures containing *P. copri*, whereas small carbohydrate molecules (DP < 9) accumulated to a greater extent compared with those in media containing only corn germ meal or *E. coli* (Fig. 6H).

We also investigated how the anti-inflammatory function is altered by increasing indole concentrations, as indole exhibits anti-inflammatory properties [57, 58]. LPS was added to SIECs following treatment with two different concentrations of indole (100 and 300 µmol/L). Although the relative mRNA expression of TNFα was unchanged, IL8 and CXCL5 were upregulated by the 300 µmol/L indole treatment (Fig. S11). Therefore, the metabolic shift in *E. coli* may contribute to improved gut homeostasis by moderating indole production and preserving substrates for bioactive AhR ligands.

*E. coli* metabolic adaptation improved gut homeostasis by reducing indole production and conserving precursors for bioactive AhR ligands. To test whether this response was specific to corn germ meal rather than a general nutrient effect, we repeated the experiment using purified starch (Fig. S12). Starch supplementation yielded a markedly different microbial community: diversity declined, Prevotellaceae did not expand, and levels of tryptophan-derived metabolites (IA, IPA, and ILA) remained low compared with corn germ meal cultures (Fig. S12A–D). Additionally, indole levels were also strongly suppressed under the starch condition, implicating corn-derived sugars in indole repression via catabolite repression (Fig. S12E). These findings highlight the specificity of corn germ meal’s complex fibre components in shaping microbiome composition and metabolic output.

Finally, we examined the relationship between Trp or indole concentrations in pig faeces and pig body weight using LC–MS/MS. The concentrations of all targeted compounds (Trp, Phe, and indole) did not change significantly with differences in pig body weight; however, Trp exhibited the largest differences with respect to body weight among the three compounds (Fig. 6I). In particular, the Trp concentration tended to be higher at higher body weights. The decrease in indole production may increase Trp availability for protein synthesis or the production of other bioactive tryptophan derivatives, potentially contributing to improved productivity.

## Discussion

The demand for livestock production continues to increase with global economic development and population growth [31, 59]. However, meeting this demand requires the sustainable utilisation of feed resources, particularly food by-products, to mitigate environmental impact. Microbial metabolism plays a key role in converting these by-products into bioavailable nutrients, underscoring the potential of dietary interventions to optimise nutrient utilisation. Dietary components can shape gut microbial communities by serving as substrates for microbial metabolism and influencing the profile of metabolites produced [60, 61]. In this study, we assessed the effects of corn germ meal supplementation on pig production by analysing its impact on microbial composition and metabolic profiles.

Using an ex vivo faecal culture model, we demonstrated dynamic shifts in microbial composition and metabolic profiles and further evaluated the bioactivity of AhR ligands using an in vitro system. Corn germ meal supplementation promoted a shift toward *Prevotella* dominance in the pig microbial community. *Prevotella* spp., a commensal bacterium of the pig microbiome [62, 63], functions as both an amino acid-associated taxon and a polysaccharide degrader, thereby contributing to amino acid metabolism. Although *Prevotella* spp. is known for its strong fibre-utilising capacity [53], and fibre supplementation is often associated with enhanced SCFA production [37–39], our metabolomic analysis showed that corn germ meal fermentation upregulated AAA metabolism, either directly or indirectly. This metabolic shift suggests that corn germ meal modulates microbial metabolic functions beyond canonical fibre fermentation pathways. Microbial metabolites derived from AAAs have been shown to regulate a wide range of biological processes, including intestinal epithelial homeostasis, immune cell function, and neuronal signalling [45, 64, 65]. These metabolites represent important mediators of host–microbiome communication at both local and systemic levels [27]. In livestock, such metabolites have been implicated in the modulation of inflammatory tone and growth performance, highlighting the potential relevance of shifts in microbial AAA metabolism for pig production [66, 67]. A key finding of this study is the identification of Trp-derived metabolites—including IA, IPA, and ILA—as ligands for the AhR in pigs. These metabolites significantly induced *CYP1A1* expression in SIECs, confirming their bioactivity through AhR activation. Our findings provide direct evidence that corn germ meal supplementation enhances microbial production of AhR ligands, potentially contributing to immunoregulation in pigs (Fig. 3). Although KyA and PPA activate AhR in humans [51, 52, 66], neither induced *CYP1A1* expression in SIECs, suggesting species-specific differences in ligand recognition, supported by structural modelling of porcine AhR (Fig. S5).

Despite the consistent predominance of *Prevotella* spp. in faecal cultures supplemented with corn germ meal across farms, the production of Trp-derived bioactive metabolites varied significantly. Farm A exhibited the highest IA and IPA levels, reaching concentrations sufficient to activate AhR, whereas farms B–D showed minimal or undetectable levels (Fig. 5 and Fig. S8). This variability suggests that farm-specific microbial communities influence the metabolic conversion of dietary fibre into bioactive compounds (e.g., differences in the abundance of bacterial species involved in AAA metabolism). In farm A, corn germ meal supplementation resulted in dominance of Prevotellaceae and Lactobacillaceae (Fig. 1B), whereas in other farms, Enterobacteriaceae, Clostridiaceae, or Lachnospiraceae were also enriched, resulting in weaker dominance of Prevotellaceae (Fig. 4A, D and Fig S7). These differences in microbial composition suggest complex cross-feeding interactions within the microbiomes. Co-culture experiments further demonstrated a shift in energy acquisition through interactions between *P. copri* and *E. coli*. Fibre degradation by *P. copri* suppressed indole production by *E. coli*, consistent with carbon source–dependent repression of tryptophan utilisation (Fig. 6F, G). Together, these findings suggest that fibre degradation by *P. copri* generates low–molecular weight carbohydrates that may alter carbon source availability for *E. coli*. Such cross-feeding interactions are consistent with catabolite repression of tryptophan utilisation, resulting in reduced indole production. In this context, *Prevotella*-mediated fibre degradation may contribute to increased tryptophan availability while limiting diversion of tryptophan toward indole biosynthesis. Bacteria preferentially utilise energy-efficient carbon sources by repressing alternative metabolic pathways [68]. In the context of corn germ meal supplementation, enrichment of monosaccharides may reduce the reliance on tryptophan as an energy substrate, thereby limiting its catabolism to indole.

Indole is a central microbial tryptophan catabolite that can be further metabolised into indoxyl sulphate, a uremic toxin in humans [69]. In addition, indole has been reported to upregulate enterotoxin expression in *C. perfringens* [70], and its production may divert tryptophan away from alternative microbial pathways that generate AhR ligands such as IPA and ILA [46]. In defined microbial communities, fibre degradation has been shown to influence tryptophan metabolism through cross-feeding of monosaccharides to *E. coli*, resulting in catabolite repression and reduced indole production [46]. In this context, our findings suggest that monosaccharides released from corn germ meal by *Prevotella* spp. may similarly suppress indole production in *E. coli*, thereby modulating the balance of microbial tryptophan metabolism. From a livestock perspective, excessive indole accumulation may be undesirable, given its reported associations with pathogenicity, metabolic toxicity, and inefficient utilisation of dietary tryptophan. Notably, certain *Bifidobacterium* species prevalent in the human gut possess both the tryptophan synthase β subunit and aromatic lactate dehydrogenase, enabling the conversion of indole into ILA via tryptophan [71]. Together with our findings, these observations underscore the metabolic flexibility of microbial tryptophan pathways within complex and diverse microbial ecosystems.

Indole, a Trp catabolite, is an intercellular signalling molecule that functions similarly to a quorum-sensing signal [72, 73]. Although indole can have detrimental effects, such as forming uremic toxins [74], it also promotes gut barrier function [57], complicating its role in gut health. These contradictory effects make it difficult to evaluate whether indole production should be restricted or promoted. In this study, we assessed the anti-inflammatory function of indole at concentrations resembling those found in the culture medium to determine its potential role in pig growth. Our findings show that indole enrichment induced pro-inflammatory cytokines in the presence of LPS in SIECs (Fig. S11). Therefore, excessive indole accumulation can have detrimental effects on livestock health. Additionally, indole and its derivatives contribute to faecal odour in livestock. Hence, modulating microbial fibre metabolism to reduce indole production could provide practical benefits for odour management in pig farming, ultimately improving environmental conditions in pig facilities.

Our findings suggest that *Prevotella*-mediated fibre degradation enhances Trp availability while simultaneously suppressing *E. coli-*derived indole production via catabolite repression. This metabolic shift may contribute to improved gut health by promoting the production of bioactive AhR ligands over potentially harmful Trp catabolites. Pectin supplementation in feed increases IPA [75] and downregulates inflammatory factors, which may operate through a similar mechanism. These results underscore the intricate interplay between microbial metabolism and host physiology, suggesting that dietary interventions aimed at modulating microbial cross-feeding interactions can have significant implications for animal health.

The presence of corn fibre consistently downregulated indole production across farms, leading to increased Trp bioavailability. Given that Trp is the second limiting amino acid in corn, its conservation is essential for optimal pig growth [76]. In addition, Trp availability influences central serotonin synthesis, which is known to regulate voluntary feed intake [77]. Although these findings were obtained in an ex vivo system, the observed reduction in microbial diversion of Trp toward indole suggests a potential increase in host-accessible Trp, providing a plausible mechanistic link between microbial metabolism and livestock production–relevant outcomes. These observations suggest that modulation of microbial Trp metabolism by corn germ meal may have practical relevance for livestock production. Although indole suppression was reproducible across farms, the induction of AhR ligands such as IA and IPA varied substantially among farms, highlighting the importance of microbiome-dependent effects when evaluating dietary interventions. It should also be noted that pigs from farms A–D differed in age because each farm operated at a distinct production stage; therefore, in this study, age was considered part of the farm-specific context rather than an experimental variable. Notably, the consistent microbial and metabolic trends observed across these farms indicate that the effects of corn germ meal supplementation were robust to baseline age differences. Together, these findings emphasize the potential value of corn germ meal as a feed ingredient that supports the retention of essential amino acids through modulation of microbial metabolism, rather than relying solely on dietary supplementation to compensate for nutritional limitations. Such strategies may contribute to improved sustainability in animal farming. As the number of independent faecal inocula was limited, this study should be interpreted as a discovery-oriented and hypothesis-generating analysis rather than a population-level quantitative assessment.

Overall, our findings reveal the potential of using corn germ meal to supplement current livestock feed and maintain or improve pig productivity. Further investigation in pigs is necessary to fully characterise the benefits of corn germ meal supplementation. Although corn germ meal enhanced the production of Trp-derived metabolites in our ex vivo system, its practical applicability requires careful consideration. In vivo, much of the protein and starch contained in corn germ meal would be digested and absorbed in the small intestine, and only a fraction would reach the large intestine. In this study, undigested corn germ meal was used intentionally to evaluate the maximal microbial metabolic potential. Therefore, the metabolite profiles observed here represent an upper-bound estimate rather than a direct reflection of in vivo fermentation. In practical feeding applications, lower substrate availability should be expected, and strategies such as enzymatic pre-treatment or fibre-rich fractionation may be necessary to achieve similar metabolic effects. Moreover, microbial conversion of tryptophan into skatole is a major cause of boar taint in pigs [78]. The suppression of indole production by corn germ meal in our ex vivo system suggests that similar dietary interventions may also influence skatole-producing pathways, although in vivo studies will be required to confirm this possibility.

The concentration of AhR ligands in the cell-based assay was determined based on previous studies, and we consider this concentration reasonable in relation to the levels observed in the ex vivo faecal culture medium. However, the exact concentration of AhR ligands in vivo remains unclear. Although our findings suggest that corn germ meal supplementation can induce AhR ligands at levels sufficient to enhance anti-inflammatory functions in pigs, direct evidence from in vivo studies is warranted to confirm this effect. Nonetheless, our study provides valuable insights into microbial and metabolic dynamics and serves as an important preliminary screening step before conducting animal trials.

## Conclusion

This study demonstrated that supplementation with corn germ meal, a fibre-rich by-product of corn starch processing, reshapes the pig gut microbiome and its metabolic outputs in ex vivo faecal cultures. Corn germ meal increased microbial diversity and enriched fibre-degrading Prevotellaceae, driving a shift toward the production of tryptophan-derived metabolites such as IA, IPA, and ILA. These compounds functioned as AhR ligands and attenuated pro-inflammatory responses in porcine intestinal epithelial cells, establishing a clear mechanistic link between dietary fibre fermentation and host immune regulation. *Prevotella*-mediated fibre degradation also enhanced tryptophan availability while repressing *E. coli-*derived indole formation through catabolite repression, thereby further promoting beneficial AhR ligand synthesis. This metabolic shift, characterised by reduced indole accumulation and enhanced Trp availability, may increase the production of AhR ligands with potential relevance for intestinal barrier function and antiviral defence in pigs [79, 80].

However, the magnitude and composition of these microbial and metabolic responses differed among farms, reflecting baseline microbiome variability and representing an important limitation when extrapolating these findings to diverse production settings. Farm-to-farm comparisons revealed that these microbial and metabolic responses vary according to baseline community structure. Understanding how dietary inputs shape microbial metabolism is essential for linking nutrition to host health, yet the capacity of food by-products to generate functional metabolites remains underexplored. By demonstrating that corn germ meal modulates microbial tryptophan metabolism and supports the formation of immunomodulatory AhR ligands, this work provides both a mechanistic foundation and preclinical evidence for subsequent in vivo validation. Collectively, these findings indicate that corn germ meal can serve as a functional feed ingredient that supports efficient utilisation of food industry by-products. By modulating microbial metabolism to conserve essential amino acids and generate bioactive metabolites, corn germ meal supplementation may contribute to improved feed efficiency and enhanced sustainability in pig production systems. Moreover, the ex vivo fermentation framework used in this study provides a practical and accessible approach for pre-evaluating the functional impacts of feed ingredients prior to in vivo validation, thereby facilitating the efficient screening of sustainable feed resources.

## Supplementary Information

Additional file 1: Supplementary Materials and Methods. (1) 16S rRNA gene amplicon sequencing and bioinformatic analysis. (2) Metabolomic analysis and faecal metabolite quantification by CE-TOF-MS and HPLC. (3) Gene expression analysis in bacterial cells by RT-qPCR. (4) Quantification of bacterial gene copy numbers by qPCR. (5) Structural analysis and homology modelling of the aryl hydrocarbon receptor (AhR).

Additional file 2: Fig. S1. Growth curves of faecal microbiota with or without corn residue supplementation. Fig. S2. Microbial composition and diversity in faecal fermentation broth in response to corn residues. Fig. S3. Beta diversity and taxonomic composition of faecal fermentation broth (farm A) in response to corn residue supplementation. Fig. S4. Amino acid metabolism and SCFA production following corn residue fermentation. Fig. S5. Structural differences in aryl hydrocarbon receptor (AhR) between humans and pigs. Fig. S6. Inter-farm variability in microbial composition and alpha diversity in response to corn residue supplementation. Fig. S7. Corn residue induces enrichment of *Prevotella* spp. Fig. S8. Farm-specific variability in the production of tryptophan-derived metabolites following corn residue supplementation. Fig. S9. *Prevotella* spp. contribute to enriched tryptophan availability. Fig. S10. Indole production depends on tryptophanase (TnaA), and *Prevotella copri* does not contribute to indole synthesis. Fig. S11. Modulatory effects of indole on pro-inflammatory cytokine and chemokine expression in swine intestinal epithelial cells. Fig. S12. Impact of starch supplementation on microbial composition in pig faecal cultures from farm A.

Additional file 3: Table S1. Chemical composition of corn germ meal used in this study. Table S2. Amino acid composition of corn germ meal and fermentation medium. Table S3. Overview of faecal samples and experimental conditions for ex vivo fermentation. Table S4. Alpha diversity indices of faecal microbiota across farms with or without corn germ meal supplementation. Table S5. Relative abundance of major bacterial taxa in faecal fermentation cultures. Table S6. Differentially abundant metabolites detected by CE-TOF-MS following corn germ meal supplementation. Table S7. Presence or absence of key aromatic amino acid-metabolising bacterial species in faecal cultures. Table S8. Summary of aromatic compounds tested for AhR activation in swine intestinal epithelial cells. Table S9. Primer sequences used for quantitative PCR analysis.

## Data Availability

16S rRNA amplicon sequence data are deposited in the NCBI BioProject database: PRJNA1224538 (https://www.ncbi.nlm.nih.gov/sra/PRJNA1224538). All data generated or analysed during this study are included in this published article and its supplementary information files. The supplementary data have not been published or submitted to any other journal.

## Abbreviations

AAA: Aromatic amino acid
AhR: Aryl hydrocarbon receptor
CE-TOF-MS: Capillary electrophoresis-time-of-flight mass spectrometry
CFU: Colony-forming units
CYP1A1: Cytochrome P450 family 1 subfamily A member 1
IA: Indole-3-acetic acid
ILA: Indole-3-lactic acid
IPA: Indole-3-propionic acid
Kyn: Kynurenine
KyA: Kynurenic acid
LC–MS/MS: Liquid chromatography–tandem mass spectrometry
LPS: Lipopolysaccharide
OD: Optical density
PLA: Phenyllactic acid
PPA: Phenylpropionic acid
PPARγ: Peroxisome proliferator-activated receptor gamma
SCFA: Short-chain fatty acid
SCM: Swine colon medium
SIEC: Swine intestinal epithelial cell
Trp: Tryptophan
