# Supplementary material for: Dynamics of tryptophan metabolites and microbial adaptations during corn by-product fermentation in the pig gut microbiome
Source: J Anim Sci Biotechnol. 2026 Feb 18;17:32. doi: 10.1186/s40104-026-01364-4 (PMC12914897; doi:10.1186/s40104-026-01364-4)
Supplement: Supplementary file 1 — Additional file 1: Supplementary Materials and Methods. (1) 16S rRNA gene amplicon sequencing and bioinformatic analysis. (2) Metabolomic analysis and faecal metabolite quantification by CE-TOF-MS and HPLC. (3) Gene expression analysis in bacterial cells by RT-qPCR. (4) Quantification of bacterial gene copy numbers by qPCR. (5) Structural analysis and homology modelling of the aryl hydrocarbon receptor (AhR). [file 40104_2026_1364_MOESM1_ESM.pdf]

Supplemental data

Dynamics of tryptophan metabolites and microbial adaptations during corn by-product fermentation in the pig gut microbiome

Additional file 1: Supplementary Materials and Methods.

Supplemental Materials and methods

1. 16S rRNA gene amplicon sequence

The 16S rRNA gene amplicon sequencing library was constructed following the manufacturer's protocol (Illumina, San Diego, CA, USA). To ensure accurate quantification of DNA amplicons, the concentration of PCR products was measured using the Qubit dsDNA HS Assay Kit (Thermo Fisher Scientific, Waltham, MA, USA). Sequencing was performed on the Illumina MiSeq platform using a 600-cycle MiSeq reagent kit, with the inclusion of internal controls (PhiX control V3; Illumina, Tokyo, Japan) for quality assurance. The resulting paired-end raw FASTQ files were processed using the QIIME2 platform (version 2023.2) (doi.org/10.1038/s41587-019-0209-9). For sequence quality control, the DADA2 pipeline was employed to denoise, merge paired reads, construct an amplicon sequence variant (ASV) table, and remove chimeric sequences. Taxonomic classification of ASVs was performed using two independent approaches. First, classifications were assigned using the Silva 138 reference database at 99% sequence similarity within the QIIME2 framework (doi.org/10.1093/nar/gks1219). Second, ASV sequences were converted to FASTA format and subjected to species-level taxonomic identification using the standalone BLAST+ software (version 2.6.0+) and the NCBI 16S_ribosomal_RNA database (last modified 2024-08-27).

2. Metabolomic analysis and faecal metabolite quantification

Quantitative assessment of microbial metabolites was also performed using CE-TOF-MS. To prepare the faecal culture medium for analysis, samples were filtered through a 5 kDa molecular weight cutoff tube, followed by extraction via vigorous shaking in the presence of 1 mM internal standards (ISs). The resulting mixture was centrifuged, and the supernatant was subjected to centrifugal filtration following the manufacturer’s protocol. Detected peaks were processed using automatic integration software (MasterHands, Keio University, Japan), extracting the peak information, including mass-to-charge ratio (*m/z*), migration time (MT), and peak area. Metabolite annotation was performed by matching MT values from capillary electrophoresis with m/z values from TOF-MS against the HMT metabolite database. Annotation criteria included a tolerance of ±0.5 min for MT and ±10 ppm for *m/z*. Relative peak areas were calculated and statistically compared between groups using Welch’s *t*-test. Metabolite concentrations were normalised relative to the IS concentration.

Short-chain fatty acids (SCFAs), including acetic acid, propionic acid, butyric acid, succinic acid, and lactic acid, were quantified in the culture medium using high-performance liquid chromatography (HPLC, Shimadzu, Kyoto, Japan), as previously described Tsujikawa *et al.*, 2024 [1], with some modifications. For sample preparation, the culture medium was centrifuged at 22,000 × *g* for 20 min at 4 °C. The resulting supernatant was filtered through a 0.45-μm PTFE membrane filter and transferred into a 0.4-mL vial (GE Healthcare Technologies, Inc., Chicago, IL, #UN503NPUORG), which was then sealed and stored at 4 °C. Prior to injection, 1 mL of the supernatant was treated with sulphuric acid using established protocols [44]. HPLC was conducted with a 10 μL injection volume

using an Aminex HPX-87H column (300 mm × 7.8 mm; Bio-Rad, Hercules, CA) equipped with a Cation H Cartridge (Bio-Rad) as a guard column. The separation was performed at 65 °C with a 5 mM sulphuric acid mobile phase at a flow rate of 0.6 mL/min. A refractive index detector, with a total run time of 70 min, was used for detection.

3. Gene expression analysis for bacterial cells

RNA Extraction from E. coli and P. copri

In total, 500 µL of *E. coli* and *P. copri* culture medium was collected, and bacterial pellets were mixed with 500 µL of RNAProtect Bacteria Reagent (1018380, QIAGEN). The stabilised pellets were enzymatically lysed for 60 min in a lysozyme solution (15 mg/mL TE buffer; Sigma-Aldrich, USA) and mutanolysin (25 units TE buffer; Sigma-Aldrich). Additionally, proteinase K treatment (1.5 mg total; 19131, QIAGEN) was performed for 30 min. The lysate was then purified using the RNeasy Mini Kit (74104, QIAGEN) following the manufacturer's protocol. To synthesise cDNA, 100 ng of total RNA was reverse-transcribed using the PrimeScript™ RT reagent Kit with gDNA Eraser (Perfect Real Time) (Takara Bio, Shiga, Japan) with random hexamer primers, according to the manufacturer's protocol. The synthesised cDNA was diluted with nuclease-free water and stored at −30 °C.

qPCR Analysis

qPCR was performed using 2.5 µL of cDNA, 1 µmol/L each primer (Table S3), and the THUNDERBIRD Next SYBR qPCR Mix (Toyobo, Japan) in a CFX Connect Real-Time PCR System. The thermal cycling conditions were as follows: initial denaturation at 95 °C for 30 s, followed by 40 cycles of denaturation at 95 °C for 10 s, annealing at primer-specific temperatures for 30 s, and extension at 72 °C for 30 s. To normalise *tnaA* expression in *E. coli* and account for variations in cDNA input among samples, the 16S rRNA gene of Enterobacteriaceae was used as an internal control, amplified using the primers Enterobacteriaceae_rplP 1F and Enterobacteriaceae_rplP 185R (Table S3).

4. Quantification of bacteria cell number

Bacterial DNA was extracted from 300 µL of ex vivo faecal culture or bacterial culture medium using the GenCheck DNA Extraction Kit [Type S/F] (Fasmac Co., Ltd.) following the manufacturer's instructions. The extracted DNA was further purified using the Monarch Spin PCR & DNA Cleanup Kit (5 µg) (#T1130S, NEB). qPCR was performed using 2.5 µL of cDNA, 1 µmol/L of each primer (Table S3), and the THUNDERBIRD Next SYBR qPCR Mix (Toyobo, Japan) in a CFX Connect Real-Time PCR System. Standard curves for Enterobacteriaceae and *Prevotella* spp. were constructed using 10-fold serial dilutions of *E. coli* and *P. copri* genomic DNA (10^3–10^8 copies). Each standard curve reaction was performed in triplicate, and amplification efficiency along with the coefficient of determination (R^2) was calculated to ensure assay reliability. These standard curves were used to determine the absolute gene copy number in faecal culture samples. Since the copy number of target

genes may not be strictly single-copy in all bacteria, we refer to these values as gene copy numbers rather than cell counts.

5. Structure of AhR and homology modelling

Cryo-EM structure of human AhR in complex with indirubin (PDB ID 7ZUB) and predicted structure of porcine AhR from AlphaFoldDB (A0AA0A1ERS1) were superimposed using PyMOL (Schrödinger, Inc.). Since indirubin has some structural analogy with the ligands of interest here, chemical model of tryptophan and kynurenine was pair-fitted to indirubin so as the benzene rings superimpose. The surrounding residues were then examined to interpret the discrepancy in ligand affinity.

Reference

[1] Tsujikawa Y, Nishiyama K, Namai F, Imamura Y, Sakuma T, Saha S, et al. Establishment of porcine fecal-derived ex vivo microbial communities to evaluate the impact of livestock feed on gut microbiome. Biosci Microbiota Food Health. BMFH Press (2024) ;43:100–9. https://doi.org/10.12938/BMFH.2023-085.
