# Supplementary material for: Dynamics of tryptophan metabolites and microbial adaptations during corn by-product fermentation in the pig gut microbiome
Source: J Anim Sci Biotechnol. 2026 Feb 18;17:32. doi: 10.1186/s40104-026-01364-4 (PMC12914897; doi:10.1186/s40104-026-01364-4)
Supplement: Supplementary file 2 — Additional file 2: Fig. S1. Growth curves of faecal microbiota with or without corn residue supplementation. Fig. S2. Microbial composition and diversity in faecal fermentation broth in response to corn residues. Fig. S3. Beta diversity and taxonomic composition of faecal fermentation broth (farm A) in response to corn residue supplementation. Fig. S4. Amino acid metabolism and SCFA production following corn residue fermentation. Fig. S5. Structural differences in aryl hydrocarbon receptor (AhR) between humans and pigs. Fig. S6. Inter-farm variability in microbial composition and alpha diversity in response to corn residue supplementation. Fig. S7. Corn residue induces enrichment of Prevotella spp. Fig. S8. Farm-specific variability in the production of tryptophan-derived metabolites following corn residue supplementation. Fig. S9. Prevotella spp. contribute to enriched tryptophan availability. Fig. S10. Indole production depends on tryptophanase (TnaA), and Prevotella copri does not contribute to indole synthesis. Fig. S11. Modulatory effects of indole on pro-inflammatory cytokine and chemokine expression in swine intestinal epithelial cells. Fig. S12. Impact of starch supplementation on microbial composition in pig faecal cultures from farm A. [file 40104_2026_1364_MOESM2_ESM.pdf]

Supplemental data

Dynamics of tryptophan metabolites and microbial adaptations during corn by-product fermentation in the pig gut microbiome

Additional file 2: Supplementary Figures (Figs. S1–S12).

Supplemental Figures

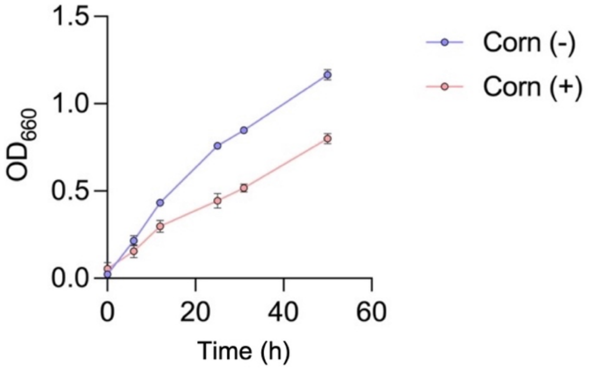

Fig. S1 Growth curves of faecal microbiota with or without corn residue supplementation
Optical density (OD_{660}) measurements of faecal cultures during a 50-hour incubation period. Faeces from the same pig (farm A) were cultured in SCM broth with or without corn residue, and OD values were measured at indicated time points. Each data point represents the mean ± SD (*n* = 3 per faecal inoculum).

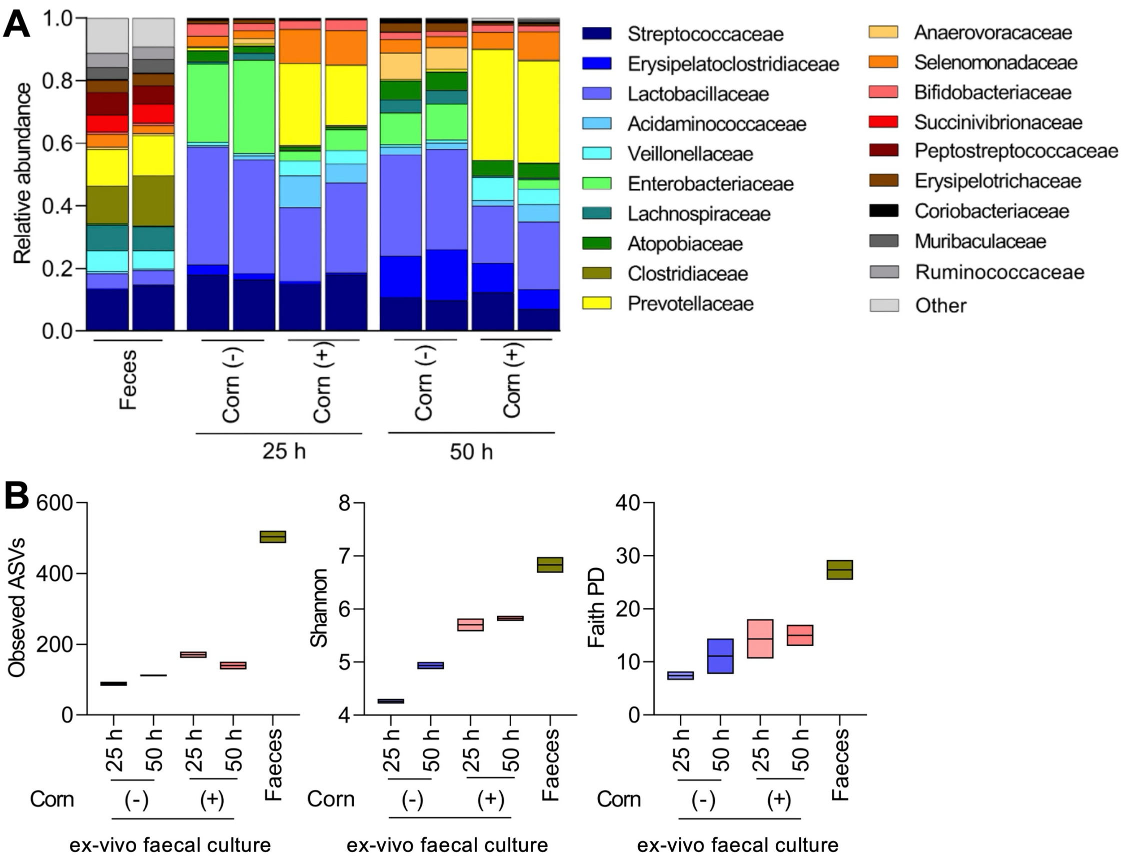

Fig. S2 Microbial composition and diversity in faecal fermentation broth in response to corn residues

(A) Relative abundance of bacterial families detected in faecal samples and faecal culture medium with or without corn residue at 25 and 50 hours. Cultures were performed in triplicate for each treatment condition (technical replicates, *n* = 2 per faecal inoculum).

(B) Alpha diversity indices (Observed ASVs, Faith's PD, and Shannon index) of microbial communities in control and corn residue-supplemented cultures at 25 and 50 h. Each data point represents a technical replicate (*n* = 2 per faecal inoculum), and horizontal bars indicate the median. Data were obtained from two representative time points (25 h and 50 h) using ex vivo cultures of faeces from a single pig (Farm A), as shown in Fig. S1.

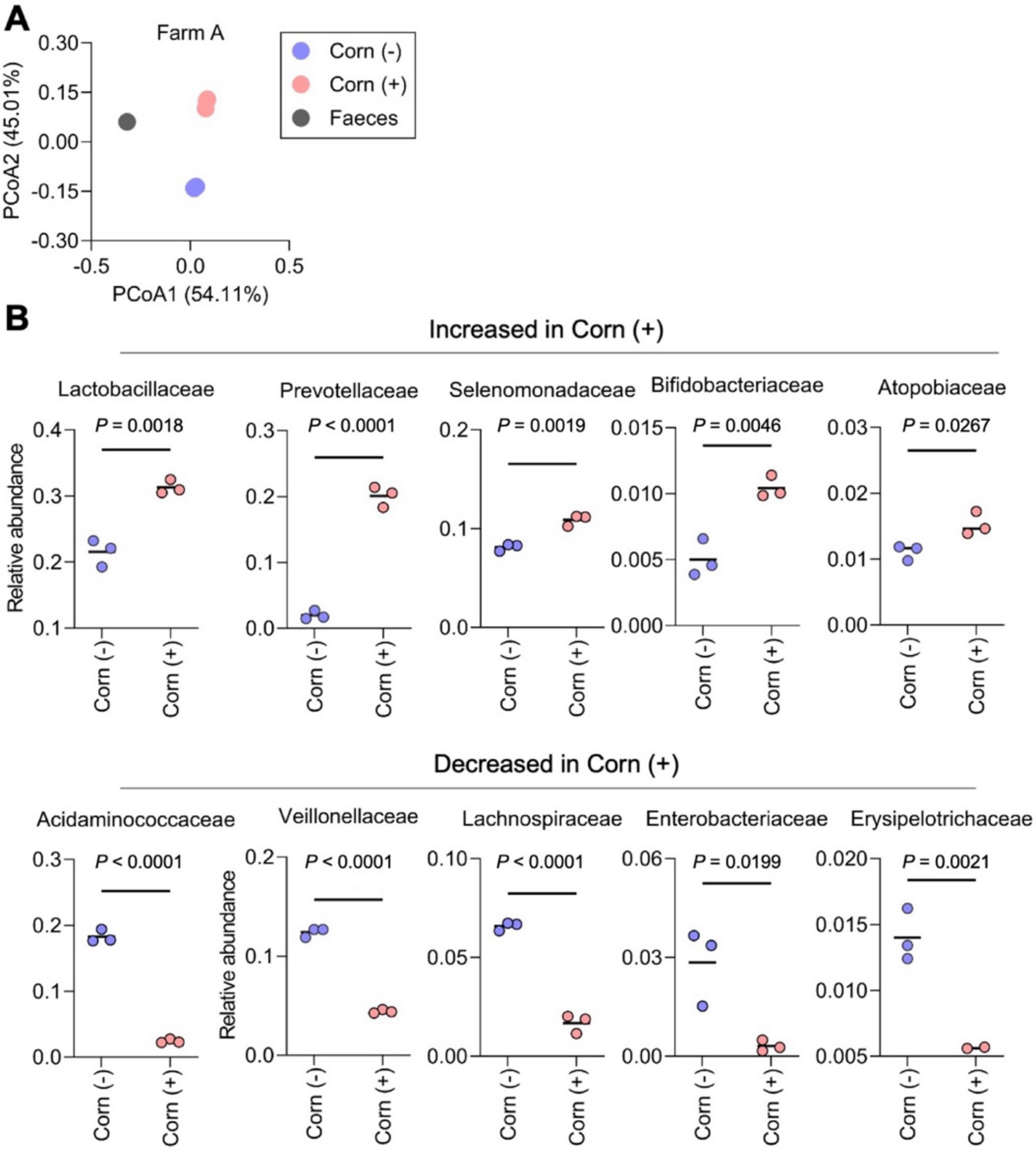

Fig. S3 Beta diversity and taxonomic composition of faecal fermentation broth (Farm A) in response to corn residue supplementation

(A) Principal coordinate analysis (PCoA) based on unweighted UniFrac distances illustrating microbial community structure shifts in faecal culture medium with or without corn residue supplementation.

(B) Relative abundance of bacterial families with ≥1% mean abundance in either treatment condition (with or without corn residue supplementation) in faecal culture medium. Taxonomic differences between groups were statistically evaluated, and significantly enriched or depleted taxa were identified based on an unpaired *t*-test (mean, $n = 3$ per faecal inoculum, $P < 0.05$).

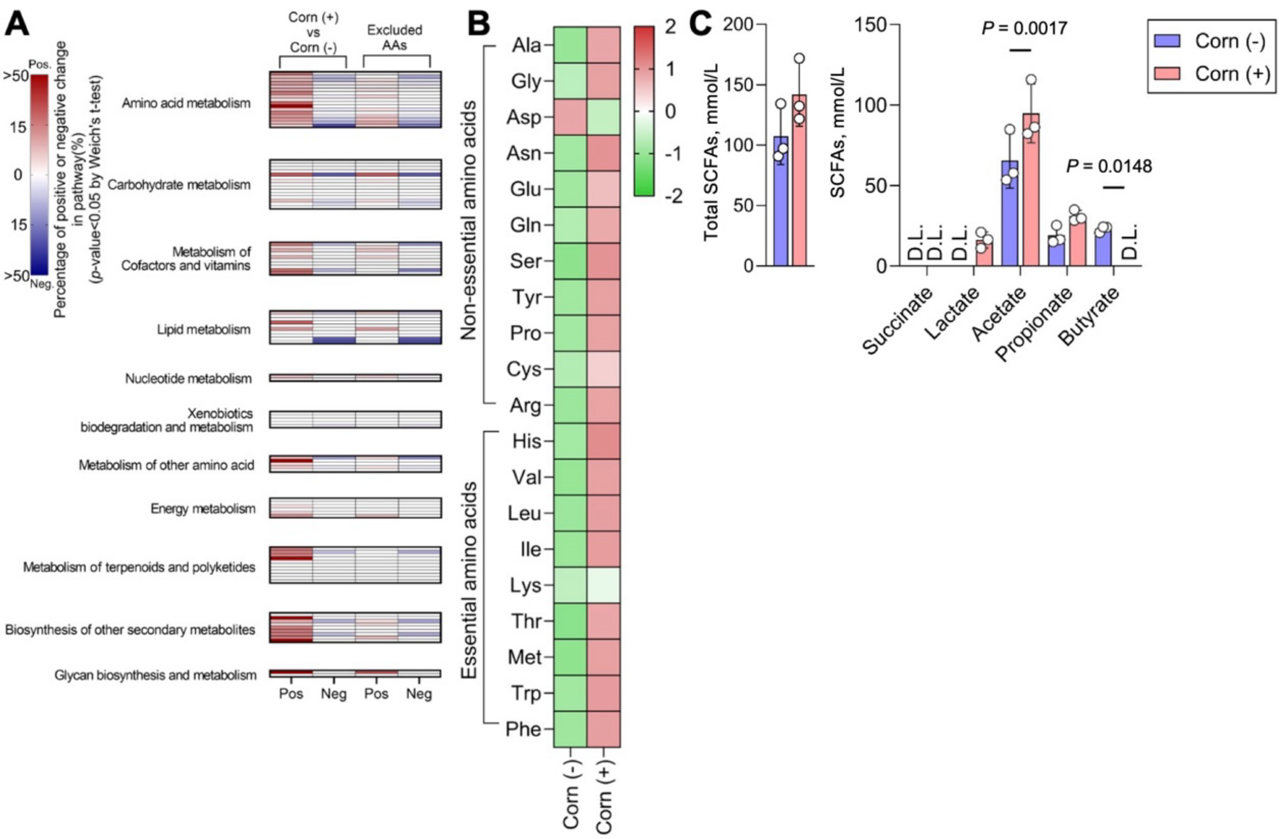

Fig. S4 Amino acid metabolism and SCFA production following corn residue fermentation

(A) Metabolic pathway analysis based on compounds detected by CE-TOFMS. Each cell represents a metabolic pathway classified based on the KEGG Pathway Database, with a heatmap indicating the proportion of metabolites that exhibit significant positive or negative changes ($P < 0.05$) in response to corn residue supplementation (mean, $n = 3$ per faecal inoculum). Statistical significance was determined using Welch's *t*-test.

(B) Heatmap representation of amino acid concentrations (intensity levels) in faecal culture medium with or without corn residue, detected by CE-TOFMS, highlighting significant metabolic shifts in amino acid profiles.

(C) Quantification of total SCFA concentrations (left) and individual SCFA levels (right) in faecal culture supernatants with or without corn residue, measured by HPLC. Each dot represents an independent biological replicate. Statistical significance was evaluated using an unpaired *t*-test for total SCFAs and a two-way ANOVA followed by Bonferroni's post hoc test for individual SCFAs ($P < 0.05$, $n = 3$ per faecal inoculum).

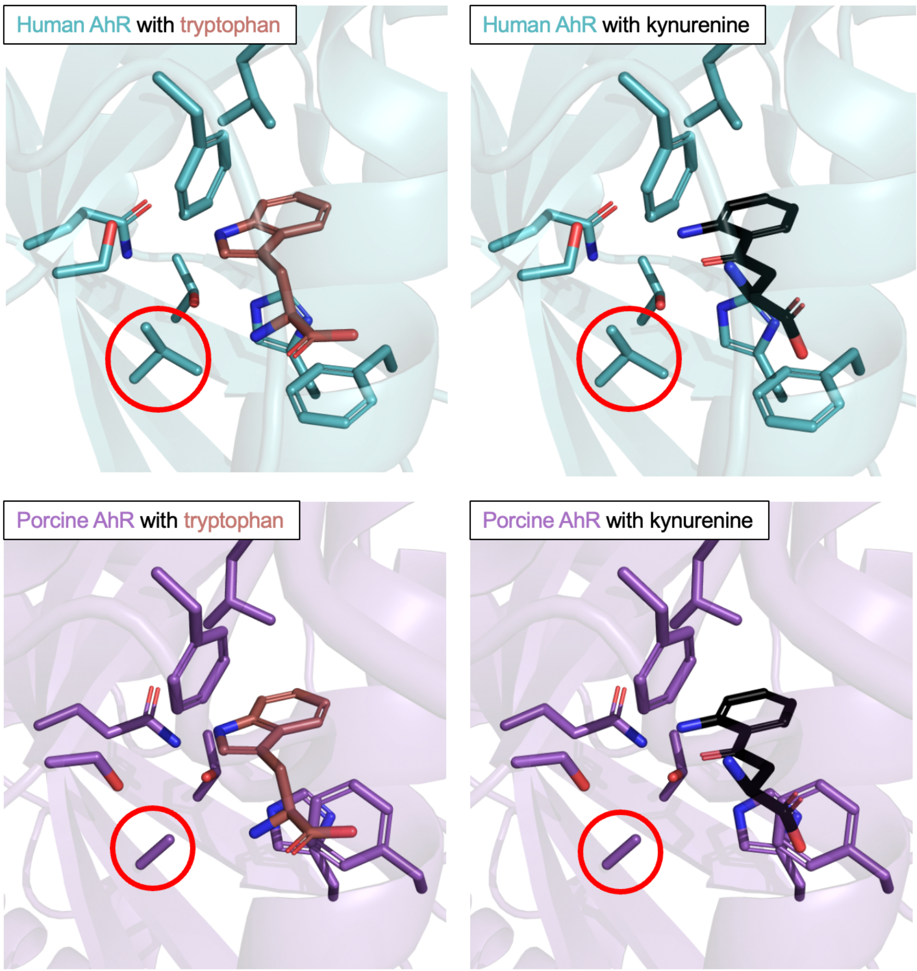

Fig. S5 Structural differences in Aryl hydrocarbon receptor (AhR) between humans and pigs

Comparative structural modelling of the AhR ligand-binding domain in humans (top) and pigs (bottom) was performed using experimental and predicted structures registered on AlphaFoldDB. Structural deviations between the two species are highlighted with red circles, indicating amino acid residue differences that may influence ligand recognition. Docking analysis with tryptophan and kynurenine suggests species-specific variations in ligand-binding affinity.

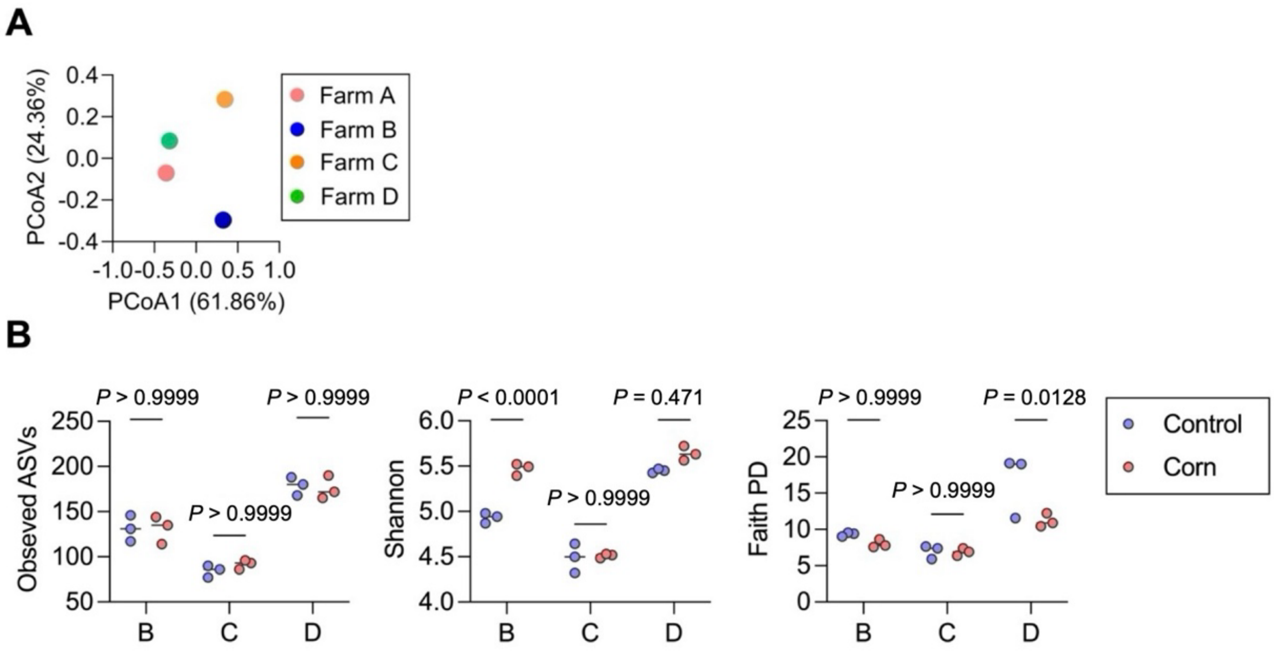

Fig. S6 Inter-farm variability in microbial composition and alpha diversity in response to corn residue supplementation

(A) Principal coordinate analysis (PCoA) based on Bray–Curtis dissimilarity illustrating differences in microbial composition among faecal samples collected from four farms (Farm A–D). Distinct clustering patterns suggest farm-specific variations in baseline microbial composition and their response to fermentation conditions.

(B) Alpha diversity indices (Observed ASVs, Faith's PD, and Shannon index) of microbial communities in control and corn residue-supplemented cultures at 50 h across different farms. Each data point represents a technical replicate, and horizontal bars indicate the median. Each treatment was conducted in triplicate using the same faecal inoculum from a single pig from each farm (n = 3 per faecal inoculum). Statistical significance was assessed using two-way ANOVA followed by Bonferroni's post hoc test ($P < 0.05$).

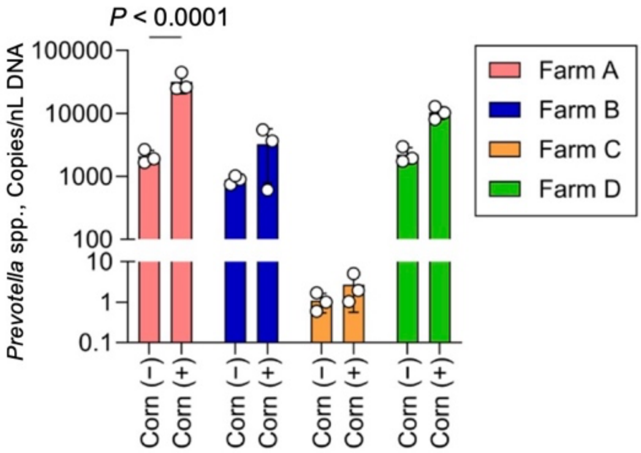

Fig. S7 Corn residue induces enrichment of *Prevotella* spp.

Quantitative PCR analysis of *Prevotella* spp. abundance in faecal culture medium with or without corn residue supplementation across four farms (Farm A–D). The copy number of *Prevotella* spp. significantly increased in Farm A following corn residue supplementation. Each dot represents an independent biological replicate. Data are presented as mean ± SD (n = 3 per faecal inoculum). Statistical significance was determined using two-way ANOVA followed by Bonferroni's post hoc test ($P < 0.05$).

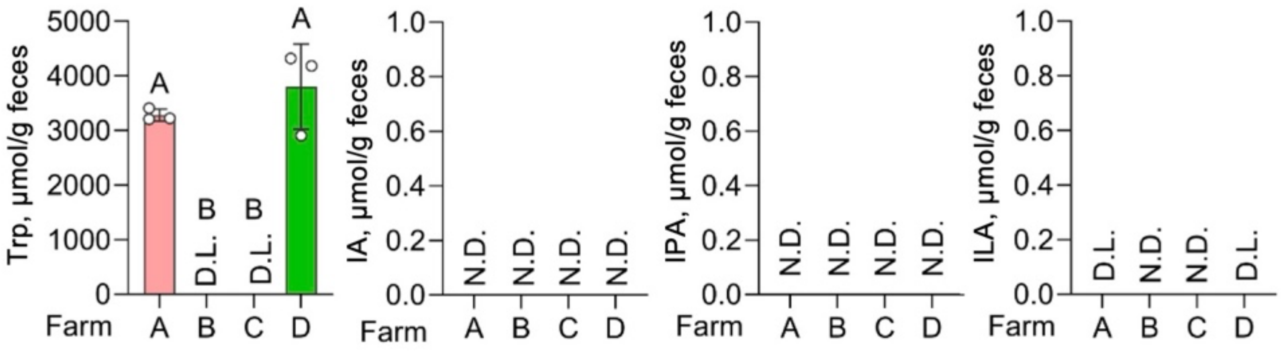

Fig. S8 Farm-specific variability in the production of tryptophan-derived metabolites following corn residue supplementation

LC-MS/MS analysis of faecal culture supernatants reveals significant variability between farms (farm A–D) in the concentrations of tryptophan and its microbial-derived metabolites, including indoleacrylic acid (IA), indolepropionic acid (IPA), and indole-3-lactic acid (ILA). Each dot represents an independent biological replicate. Data are presented as mean ± SD (*n* = 3 per faecal inoculum). Statistical significance was determined using one-way ANOVA followed by Bonferroni's post hoc test ($P < 0.05$). Different letters indicate statistically significant differences between groups.

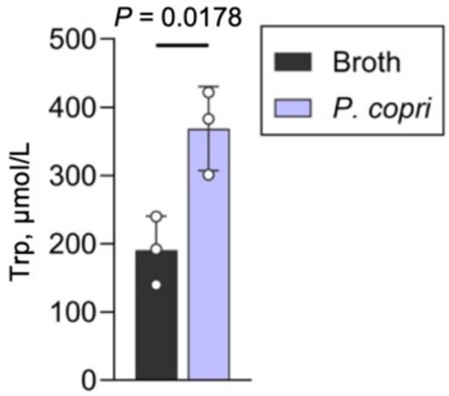

Fig. S9 *Prevotella* spp. contribute to enriched tryptophan.

LC-MS/MS quantification of tryptophan concentrations in SCM broth and *Prevotella copri* cultures. Tryptophan levels were significantly elevated in *P. copri* cultures, indicating a link between *Prevotella*-mediated fibre degradation and increased free tryptophan availability. Each dot represents an independent biological replicate. Data are presented as mean ± SD (n = 3 per condition). Statistical significance was assessed using an unpaired t-test ($P < 0.05$).

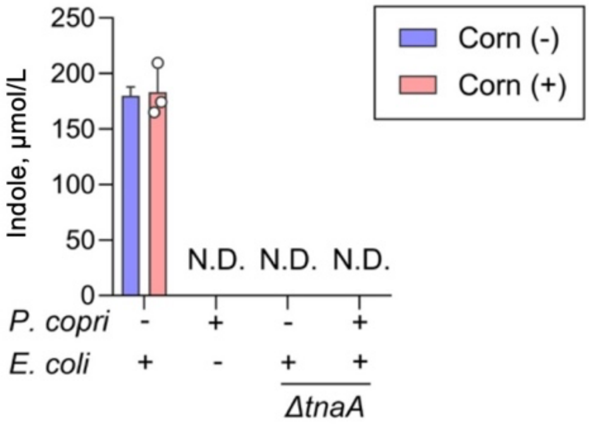

Fig. S10 Indole production depends on tryptophanase (TnaA), and *Prevotella copri* does not contribute to indole synthesis.

LC-MS/MS analysis of indole concentrations in monoculture and co-culture media demonstrates that indole production depends on wild-type *Escherichia coli*, while *E. coli ΔtnaA* and *P. copri* do not produce indole. Each dot represents an independent biological replicate. Data are presented as mean ± SD ($n = 3$ per condition). Statistical significance was determined using one-way ANOVA followed by Bonferroni's post hoc test ($P < 0.05$).

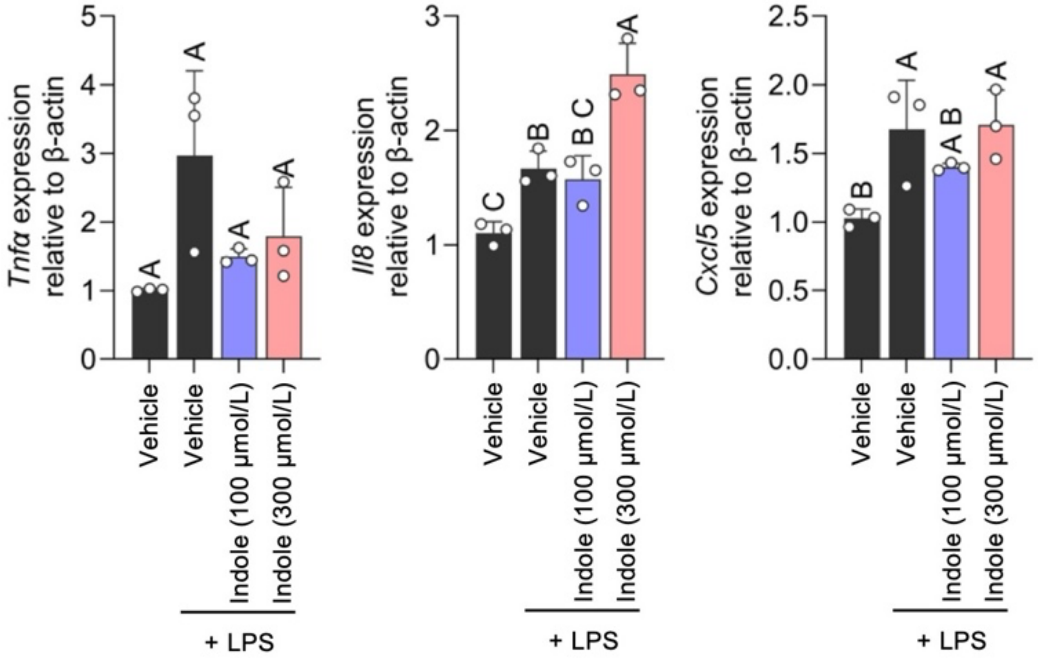

Fig. S11 Modulatory effects of indole on pro-inflammatory cytokine and chemokine expression in SIECs

Swine intestinal epithelial cells (SIECs) were stimulated with lipopolysaccharide (LPS, 1 μg/mL) following pre-treatment with indole (100 μmol/L or 300 μmol/L) or vehicle control (DMSO) for 48 hours. qPCR analysis of *TNF-α*, *IL-8*, and *CXCL5* expression reveals that while *TNF-α* levels remain unchanged, high-dose indole treatment (300 μmol/L) significantly increases the expression of *IL-8* and *CXCL5*. Data are presented as mean ± SD (n = 3 per condition). Each dot represents an independent biological replicate. Statistical significance was determined using one-way ANOVA followed by Bonferroni's post hoc test ($P < 0.05$). Different letters indicate statistically significant differences between groups.

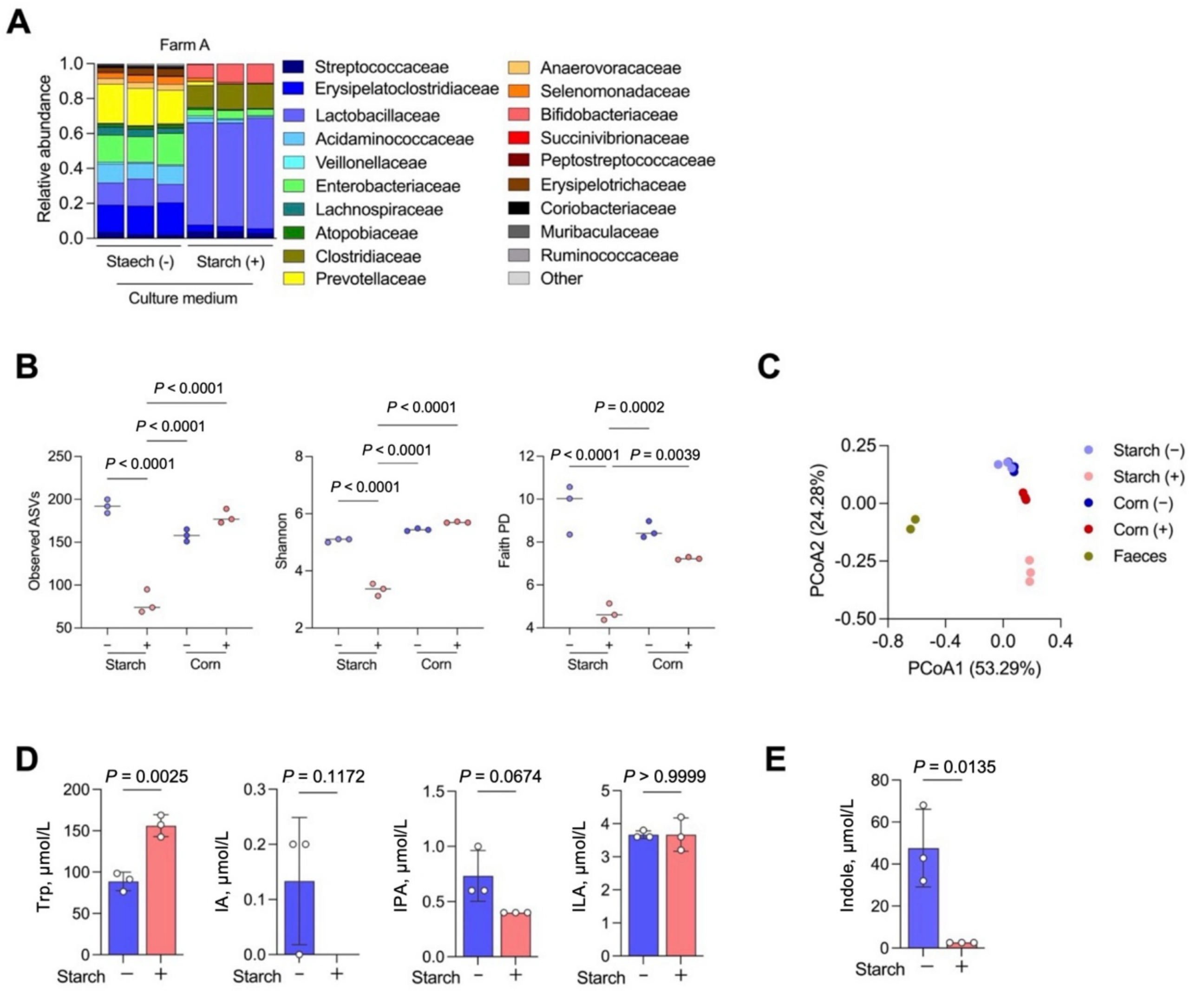

Fig. S12 Impact of starch supplementation on microbial composition in pig faecal cultures from Farm A

Pig faecal cultures were subjected to *ex vivo* fermentation assays with or without supplementation of 5% (w/v) starch for 50 hours.

(A) Relative abundance of bacterial families in original faecal samples and their corresponding cultures following fermentation in the presence or absence of starch. Each bar represents microbial composition derived from faeces of an individual pig from Farm A. Experiments were conducted in triplicate for each treatment (technical replicates, n = 3 per faecal inoculum).

(B) Alpha diversity indices, including Observed ASVs, Shannon diversity, and Faith's phylogenetic diversity (PD), in control and starch-supplemented cultures. Each point represents a technical replicate (n = 3 per faecal inoculum); horizontal bars indicate median values. Statistical significance was determined by the Mann–Whitney U test.

(C) Principal Coordinate Analysis (PCoA) based on unweighted UniFrac distances illustrating shifts in microbial community structure following starch or corn residue supplementation (data from Fig. 1)

compared to original faeces.

(D) LC–MS/MS quantification of tryptophan (Trp), indoleacrylic acid (IA), indolepropionic acid (IPA), and indole-3-lactic acid (ILA) in culture supernatants from Farm A in the presence or absence of starch. Each dot represents an independent biological replicate (*n* = 3 per faecal inoculum); data are presented as mean ± SD. Statistical significance was assessed by unpaired *t*-tests.

(E) Indole concentration measured by LC–MS/MS in faecal cultures, demonstrating significantly reduced levels with starch supplementation. Statistical significance was assessed by unpaired *t*-test.
